# Improving care for patients with *Clostridioides difficile* infection: A clinical practice and healthcare systems perspective

**DOI:** 10.3389/fmed.2022.1033417

**Published:** 2023-01-12

**Authors:** Lucy Hocking, Mark Wilcox, Nicola Petrosillo, Paul Griffin, Theodore Steiner, Gail Attara, Joel Doré, Mark Cabling, Stephanie Stockwell, Robert J. Romanelli, Sonja Marjanovic

**Affiliations:** ^1^RAND Europe, Cambridge, United Kingdom; ^2^School of Medicine, University of Leeds, Leeds, United Kingdom; ^3^Leeds Teaching Hospitals NHS Trust, Leeds, United Kingdom; ^4^Policlinico Universitario, Bio-Medico Campus University Hospital, Rome, Italy; ^5^Mater Clinical Unit, Faculty of Medicine, The University of Queensland, Brisbane, QLD, Australia; ^6^Division of Infectious Diseases, The University of British Columbia, Vancouver, BC, Canada; ^7^Canadian Society of Intestinal Research, Vancouver, BC, Canada; ^8^Gastrointestinal Society, Vancouver, BC, Canada; ^9^INRAE, MetaGenoPolis, AgroParisTech, Micalis Institute, Université Paris-Saclay, Jouy-en-Josas, France

**Keywords:** *Clostridium difficile*, *Clostridioides difficile*, *C. difficile* infection, healthcare improvement, healthcare systems, patient pathway

## Abstract

**Introduction:**

Arriving at a *C. difficile* infection (CDI) diagnosis, treating patients and dealing with recurrences is not straightforward, but a comprehensive and well-rounded understanding of what is needed to improve patient care is lacking. This manuscript addresses the paucity of multidisciplinary perspectives that consider clinical practice related and healthcare system-related challenges to optimizing care delivery.

**Methods:**

We draw on narrative review, consultations with clinical experts and patient representatives, and a survey of 95 clinical and microbiology experts from the UK, France, Italy, Australia and Canada, adding novel multi-method evidence to the knowledge base.

**Results and discussion:**

We examine the patient pathway and variations in clinical practice and identify, synthesize insights on and discuss associated challenges. Examples of key challenges include the need to conduct multiple tests for a conclusive diagnosis, treatment side-effects, the cost of some antibiotics and barriers to access of fecal microbiota transplantation, difficulties in distinguishing recurrence from new infection, workforce capacity constraints to effective monitoring of patients on treatment and of recurrence, and ascertaining whether a patient has been cured. We also identify key opportunities and priorities for improving patient care that target both clinical practice and the wider healthcare system. While there is some variety across surveyed countries’ healthcare systems, there is also strong agreement on some priorities. Key improvement actions seen as priorities by at least half of survey respondents in at least three of the five surveyed countries include: developing innovative products for both preventing (Canada, Australia, UK, Italy, and France) and treating (Canada, Australia, and Italy) recurrences; facilitating more multidisciplinary patient care (UK, Australia, and France); updating diagnosis and treatment guidelines (Australia, Canada, and UK); and educating and supporting professionals in primary care (Italy, UK, Canada, and Australia) and those in secondary care who are not CDI experts (Italy, Australia, and France) on identifying symptoms and managing patients. Finally, we discuss key evidence gaps for a future research agenda.

## 1. Introduction

*Clostridioides difficile* (*C. difficile*), is a bacterium that commonly colonizes the human large intestine (1, 2). *C. difficile* colonization is not typically harmful, as other bacteria in the digestive system suppress its growth. However, under certain conditions, such as with the use of antibiotics or following gastrointestinal surgery (1, 2), *C. difficile* can grow in its vegetative state, producing toxins that damage the intestinal epithelium. Toxigenic *C. difficile* infection (CDI) can cause a range of bowel problems such as diarrhea, nausea and abdominal pain, and other symptoms like fever and loss of appetite (2). More severe CDI can cause complications such as pseudomembranous colitis, septic shock and death (1, 3, 4). The European Centre for Disease Prevention and Control estimates that CDI has a 4% mortality rate (5), which is higher in those who are frail, hospitalized (including in intensive care units) and/or elderly (1, 6, 7). However, the 4% mortality rate may be an underestimation given the challenges in attributing death directly to CDI. For example, 2020/21 data from England suggests that the 30-day all-cause fatality rate of CDI is closer to 13% (8).

Some aspects of the burden of CDI are relatively well understood, such as healthcare costs and mortality rates (9–12), but further research and validation is needed on the challenges faced by clinicians and patients in arriving at a CDI diagnosis, accessing treatment options and managing infections, including dealing with recurrences.

In this paper, we identify and reflect on the diverse requirements for effective clinical care for patients with CDI. As a foundation, we provide a brief overview the patient care pathway and explore variations in practice. We discuss challenges and key improvement needs as they relate to the care pathway as well as the wider healthcare system which frames clinical care. In doing so, we address the lack of multidisciplinary research that considers both clinical practice related requirements associated with diagnosis, treatment, ongoing patient monitoring, management of CDI recurrence and healthcare system influences on patient care, for example those related to access and organization of services, guidelines and regulation, and education and awareness raising (for patients and clinicians).

We focus specifically on patient care (i.e., diagnosis, treatment of initial CDI, patient monitoring and dealing with recurrence) and offer multidisciplinary and comprehensive insights drawn from a multi-method approach that tackles the often piecemeal nature in which challenges to patient care are at times researched. We consider the whole care pathway and the healthcare system that frame it. We recognize that infection prevention and control in hospital and community environments is also an important aspect of CDI management given that CDI is a frequent cause of healthcare-acquired infection (13), but this is discussed in numerous other literature and not covered in the scope of this study.

## 2. Materials and methods

This study involved conducting a narrative literature review, consultations with clinical experts and patient representatives, and a survey of clinical experts that sought to inform priorities for improvement in practice and key evidence gaps in need of further research.

The study focused on reviewing evidence from high-income countries (HICs), with a particular emphasis on the United Kingdom (UK), Italy, France, Canada, and Australia. These countries were selected given their geographical variety and all having a public healthcare system free at the point of service.

### 2.1. Narrative review

We conducted a narrative review following principles of rapid evidence assessment (REA) methodology (14). This includes: (1) development of a systematic search strategy, inclusion and exclusion criteria, and running the literature search; (2) screening the titles and abstracts of articles against the criteria; (3) prioritizing articles for inclusion in consideration of topic coverage, comprehensiveness, geographical focus and publication year; and (4) a full-text review and analysis of prioritized articles.

Two searches in PubMed were conducted in May 2021. The first focused on identifying relevant literature from the five case example countries (Australia, Canada, France, Italy, and the UK) and covered a 10-year timeframe (2011–2021). We also conducted a supplementary second search to identify additional literature from HICs more widely and focused on most recent review articles from the past 5 years (2016–2021), to fill gaps in literature on case example countries. A web-based gray literature search (June 2021) complemented the academic literature search and helped identify regulations and guidelines on CDI patient care in the case example countries. Twenty-nine papers were included (see Supplementary material for PRISMA flow diagram). We also considered some additional publications on specific points of detail raised in the literature that is included in the narrative review, where this was merited to provide further clarity on specific issues related to context or updates in regulation for example.

### 2.2. Consultations with clinical experts and patient representatives

We conducted consultations with leading CDI clinical experts and some patient representatives from the case example countries to refine, nuance and enrich insights from the literature and address gaps in the existing evidence base. This allowed us to gain valuable experiential knowledge of diagnosing, treating and managing CDI and associated challenges. Consultations took the form of in-depth, semi-structured interviews and structured workshops (August – December 2021). With informed consent, eight one-to-one semi-structured interviews were carried out by the research team (LH and SS) with three experts from Canada, and one from Australia, the UK, France, Italy and a representative of a global foundation. Interviews followed established qualitative research methods used in health services research (15). Interview evidence is referenced with Int X, with X being the code number for an individual interviewee. To preserve anonymity in some instances where there is a direct risk of identity disclosure, and in line with informed consent, we withhold an interview reference number.

In addition, over the course of three online workshops (September 2021) health services research experts from RAND Europe (SM, LH, RR, and SS) and clinical and patient representative co-authors (MW, NP, TS, PG, GA, and JD) met in small groups/individually with the research team to enable knowledge-exchange and reflection on learning from the narrative review and interviews.

### 2.3. Survey of clinical and other experts on CDI

An online survey engaged experts from the five case example countries to explore views on needed priority actions for improving the care pathway for patients with CDI. The survey was designed based on findings from the earlier narrative review and consultations, with thematic analysis informing its structure and organization. The survey had subsections on different overarching areas of improvement (diagnosis and treatment; access and organization of service delivery and quality of care; guidelines and regulations; education and awareness raising for patients; and education and awareness raising for clinicians). These themes were developed based on the narrative review and consultation data, and in discussion with clinical expert and patient representative co-authors.

Within each thematic area, as part of the survey, respondents were asked to select improvement actions which they considered most important. The number of improvement actions selected as most important was dependent on the number of actions available to select from – respondents were asked to select actions within a top third threshold. For example, if six improvement actions were available, respondents were asked to select up to two they thought were most important. Respondents were also asked to share views on the most important gaps in evidence that need to be filled to inform future research and improvement. They were also asked to provide information on the nature of the CDI patient care pathway in terms of a patient’s first point of contact with the healthcare system and in terms of referral practices (see the Supplementary material for the survey tool). The survey was disseminated via national and international networks and professional societies. It was open from January to May 2022 to allow sufficient time for respondents, many of whom were also involved in efforts to respond to the COVID-19 pandemic. All survey respondents participated with informed consent.

### 2.4. Analysis and synthesis

The findings across the narrative review and expert consultations were analyzed thematically, triangulated and synthesized by the research team to develop a multifaceted understanding of CDI diagnosis, treatment and management pathways across the countries of interest and associated challenges in patient care. This enabled us to develop the survey questions focusing on exploring priorities in terms of key areas in need of improvement in patient care. The survey was analyzed using the SmartSurvey analysis export tool and Excel, using a thematic approach. Survey analysis considers both similarities and differences in findings across the participating countries.

### 2.5. Ethics

This study involved a literature review, interviews with clinical experts and patient representatives, and a survey of clinical experts. The research was conducted in accordance with the Declaration of Helsinki. It was judged to pose minimal risks to participants and not to require ethical approval. It was reviewed retrospectively by the RAND Human Subjects Protection Committee and determined to be exempt under 45 CFR 46.104(d)(2)(ii), and, although exempt, the study’s procedures and materials were found by the committee to be consistent with all rules laid out under 45 CFR 46 for the conduct of non-exempt human subjects’ research. All participants gave informed consent and were provided with participant information sheets as part of this process.

## 3. Results

### 3.1. Survey respondents

We received 95 eligible responses to the survey. This includes 38 responses from Italy, 25 from UK, 16 from Australia, 12 from Canada, and 4 from France. While efforts were made to share the survey with relevant associations in France, some declined to engage due to CDI not being their core current focus. Given the low number of responses from France in particular, care should be taken when interpreting the survey findings presented at country level.

The majority of respondents stated infectious diseases as their primary area of work (61%), but there was also input into the survey from other clinical areas (e.g., gastroenterology and primary care) and from microbiologists. Most respondents identified as physicians/medical doctors (82%), but a variety of views were gathered, including for example from nurses (10%). See Supplementary material for further information on the demographics of respondents.

### 3.2. The clinical care pathway and associated challenges

The patient pathway for CDI involves key stages spanning diagnosis, treatment of initial CDI, patient monitoring and follow-up, and management of recurrence. Many aspects of care are similar across the case example countries, but there are also some important differences (see Tables 1–3). In this section, we discuss the main aspects of the care pathway and associated challenges to optimizing patient care, drawing on evidence from the review of the literature and consultation with experts.

**TABLE 1:** Symptom presentation and diagnosis.

Country	Which tests are conducted, and in what order?	Who conducts tests?	Who pays for diagnostic tests?	How long does it take to receive a diagnosis?
UK	Glutamate dehydrogenase (GDH) [or polymerase chain reaction (PCR) test for toxin gene] screening test plus toxin test. If GDH or PCR is negative, no toxin test is needed. If GDH or PCR is positive and toxin test is positive, this indicates CDI. Some labs may add a PCR test if GDH positive, but toxin test negative to provide infection control information.	Hospital lab.	Centrally funded.	Outpatient: 1–2 days, depending on sample transport time. Inpatient: 4–12 h, depending on lab processing.
France	Culturing to confirm if *C. difficile* is present. If this is positive GDH and EIA for toxin. A positive diagnosis can be confirmed if GDH and toxin tests are positive. If results are ambiguous, direct toxin by PCR can be conducted.	Outpatients: private labs. Inpatients: hospital or private labs.	65% of cost is centrally funded; rest to be paid by patient depends on their situation (long disease/other chronic conditions can be 100% reimbursed).	Outpatient: around 5 days. Inpatient: 2 days.
Italy	In many laboratories the confirmatory test is EIA. Some labs use NAAT for molecular detection of *C. difficile* toxin genes.	Hospital and private labs (no difference between outpatient and inpatient).	Inpatients: centrally funded. Outpatients: some out of pocket expenses for patient with the remainder covered by the national health service. Private tests paid for by patients.	Outpatient: up to 5 days (usually 2 days). Inpatient: 5 days (usually 2 days).
Canada	PCR alone or PCR and toxin testing (in any order).	Usually private labs, but can be hospital labs.	Centrally funded.	Outpatient: 1–2 days (depending on how quickly patient seeks care. The more severe cases presenting to emergency departments get diagnosed immediately). Inpatient: within 1 day.
Australia	Dependent on lab – some use PCR, followed by toxin tests if required, others still use antigen testing alone and others PCR alone.	Dependent on where patient presents - public hospital system has public lab, private hospitals use a range of private labs.	Usually funded publicly or paid for by health insurers.	1–2 days for both inpatients and outpatients.

**TABLE 2:** Treatment of initial infection, and patient monitoring and follow-up.

Country	How quickly does treatment commence?	How is treatment initiated and by whom?	Patient involvement in treatment decision-making	Treatment course for initial, mild infection	Treatment course for initial, severe infection	How long does treatment typically last?	Who pays for treatment?	How are patients monitored?	How are patients assessed for cure (if at all)?
UK	Same day as diagnosis.	Larger hospitals: specialists determine treatment regime (infectious disease specialists, gastroenterologists). Smaller hospitals: primary physician. Outpatient: primary care (possibly with advice from microbiologists based on test results).	Little involvement in initial infection. Greater involvement for recurrences (particularly FMT).	Metronidazole (or vancomycin).	Vancomycin (or fidaxomicin for high recurrence risks). Alternatives are: high dose vancomycin (with/without IV metronidazole), rifampicin or IV immunoglobulin. Life threatening infection treated with nasogastric/rectal vancomycin, with/without IV metronidazole.	10 days.	Centrally funded.	Outpatient: little-no monitoring. Inpatient: regular monitoring of stool, bowel movements and lab tests. Involves microbiologists, infectious disease specialists, infection prevention/control staff and the primary physician.	Symptom resolution.
France	Same day as diagnosis (sometimes before confirmation of diagnosis).	Generalist or practitioner.	Yes, always.	Fidaxomicin.	Vancomycin or fidaxomicin.	10 days.	65% covered for all patients; rest to be paid according to patient situation.	Mainly resolution of diarrhea.	Symptom resolution.
Italy	Same day as diagnosis	Primary physician (with GI or infectious disease specialist support if non-specialists) for in and outpatients.	Little involvement in both initial and recurrent infection.	Vancomycin or, less commonly, fidaxomicin.	Vancomycin (increasing use of fidaxomicin following updated ESCMID guidelines).	10 days.	Centrally funded.	Outpatient: little-no monitoring. Inpatient: monitored by ID/gastroenterologists (e.g., bowel movements, blood tests).	Symptom resolution.
Canada	Same day as diagnosis (for inpatient and outpatients).	Outpatient: primary care. Inpatient: primary physician (with input from pharmacist or, in fulminant/failure to respond cases, specialists).	Little involvement in initial infection. Greater involvement for recurrences (particularly FMT).	Vancomycin (can be metronidazole or fidaxomicin if available).	Vancomycin (or fidaxomicin if available). Severe and complicated infection primarily treated with vancomycin with IV metronidazole.	10–14 days (typically 10 days for the first episode).	Outpatient: out-of-pocket payment for vancomycin and fidaxomicin, but not metronidazole (although this varies by province). Inpatient: centrally funded.	Outpatient: little-no monitoring. Inpatient: regular monitoring of stool, bowel movements and lab tests. Fulminant cases involve infectious disease specialists, gastroenterologists and/or surgical staff.	Symptom resolution.
Australia	Inpatient: same day as diagnosis. Outpatient: 3–5 days.	Primary physician (with GI input in severe cases) for both in and outpatient.	Little involvement in initial infection. Greater involvement for recurrences (particularly FMT).	Metronidazole (or vancomycin, fidaxomicin).	Vancomycin with IV metronidazole is first line, second line is nasogastric vancomycin and IV metronidazole, with/without rectal vancomycin. FMT in refractory infection.	10–14 days	Subsidized by Medicare.	Outpatient: little-no monitoring. Inpatient: regular monitoring of bowel movements and lab tests. Ideally involved infectious disease specialists.	Symptom resolution.

**TABLE 3:** Preventing and managing recurrences.

Country	What are the approaches for preventing recurrences?	How are recurrences diagnosed?	What is the treatment course for initial recurrence?	What is the treatment course for 2+ recurrences?	Who pays for treatment for recurrences?
UK	Fidaxomicin or monoclonal antibody therapy (although with limited use due to cost).	Same as initial infection.	Fidaxomicin (or vancomycin if cost is issue).	Fidaxomicin (if not used for initial infection); tapered/pulsed vancomycin; IV immunoglobulin; or FMT.	Centrally funded.
France	Antibiotic stewardship and specific selection of antibiotics that will minimally alter the normal anaerobic microbiota.	Same as initial infection, but faster; often with primary care physician (for outpatients).	Fidaxomicin.	Vancomycin with tapering of doses or FMT.	Antibiotics: 65%+ of fidaxomicin is centrally funded depending on coverage. For FMT, there is a different legal framework; payment is taken care of but modalities differ center by center; the assessment cost is paid for according to the centers. Depending on the commission of the establishment/hospital.
Italy	Fidaxomicin, taper/pulse regime of antibiotics, monoclonal antibody therapy.	Same as initial infection.	Generally vancomycin (increased trend of fidaxomicin if the initial treatment was done with vancomycin). If the initial treatment was done with fidaxomicin, generally bezlotoxumab is used for preventing further recurrences.	Tapered vancomycin, fidaxomicin, monoclonal antibodies, and FMT if available.	Centrally funded.
Canada	Tapered vancomycin can be used in high recurrence risks (although rarely for first episodes).	Same as initial infection or reliance on clinical presentation.	Vancomycin (very high risk patients may have tapered dose of vancomycin).	Tapered dose of vancomycin, or FMT.	Inpatient: hospital funding structure. Outpatient: the patient.
Australia	Ceasing use of contributing antimicrobials, taper regime of vancomycin, FMT.	Same as initial infection.	Vancomycin.	Vancomycin with/without taper, fidaxomicin, rifaximin chaser or FMT.	Medications are funded through the pharmaceutical benefits scheme. For inpatients, medication would be centrally funded. FMT conducted in public hospital will be covered by public hospital funding structure.

#### 3.2.1. The diagnosis pathway

The diagnosis pathway for the example countries is outlined in Table 1. Diagnosis involves deciding if a test for CDI is required based on clinical signs such as diarrhea, abdominal pain or distension, ileus, and toxic megacolon [(2, 16, 17) Int1,7]; deciding which test to use; performing the test and interpreting results. Testing is only recommended on symptomatic patients as *C. difficile* can be present in the digestive systems of healthy people (2, 4).

Where diagnosis takes places varies, in part depending on whether the patient presents to primary care physicians in the community or in hospital (including emergency department), which in turn can depend on how unwell a patient is, with more severe cases more likely to be identified in hospital (Int2, 4–7).

In most case example countries, survey data suggests that the first point of contact with the healthcare system for the majority of patients with *community acquired* CDI is a primary care professional (92% of survey respondents in the UK conveyed this to be the case, 88% in Australia, 67% in Canada and 61% in Italy). However, this was not the case for France, where 25% of respondents reported that gastroenterologist experts in an outpatient hospital setting were the primary point of contact (although only four respondents were from France). Other primary points of contact identified by survey respondents ranged from emergency care settings to community-based infection prevention and control teams. However, some survey respondents felt that there was not one predominant point of contact, and this is likely to reflect diverse practices regionally, diversity between healthcare systems and differences related to variety in patient symptoms.

For patients with *hospital acquired* CDI in Australia, Canada, the UK, and France, the first point of care for patients tends to be the person under whose care they are more generally (81, 75, 80, and 50% of survey respondents, respectively). However, in Italy, this was only seen as the most common route by 40% of survey respondents. More common in Italy was referral to an infectious disease expert (47% of survey respondents), while this option was rarer in France, Australia, the UK, and Canada (25, 19, 16, and 8%, respectively). See Supplementary material for further information.

Who the patient will be referred to from the first point of contact in a community settings seems to vary both within and between countries, with patients being referred to either gastroenterologists, infectious disease experts and more rarely emergency care settings (see Supplementary material). Onward referral will depend on factors such as the severity of patient symptoms, parts of the country and preferences and personal experiences of the referring healthcare professional.

In terms of onward referral from inpatient/hospital admission settings, in Australia, Italy, and Canada this is most often to an infectious disease specialist in the inpatient setting (69, 74, and 58%, respectively). Less common is referral to other experts such as gastroenterologist, patients receiving referrals to multiple healthcare professionals at the same or to infection prevention and control nurses/teams (see Supplementary material for further detail).

Diagnostic testing can be done in public sector facilities or by private laboratories and this can vary both within and between countries, dependent on health system service organization and capacity (Table 1). For example, in Canada and Australia, most outpatient testing is conducted by private labs (interviewee reference numbers withheld to preserve anonymity) and while most hospitals have outpatient labs many patients live closer to private labs than hospital-based ones. In Canada, CDI testing does not require out of pocket payment by patients, including to private labs, with payment covered by central government funding (interviewee reference number withheld to preserve anonymity). Across the countries considered in this research, for patients who first present with symptoms in primary care, diagnostic testing is generally ordered by primary care providers (Int4–7). For patients who present with symptoms in hospital, diagnosis is generally overseen by hospital staff and specialists, such as infectious disease specialists and/or gastroenterologists (Int5–6).

The main diagnostic methods for CDI testing in patients of all ages are enzyme immunoassays [EIAs, to detect A/B toxin or the glutamate dehydrogenase (GDH) enzyme produced by *C. difficile*] and nucleic acid amplification tests (NAATs), with toxigenic culture and cell cytotoxicity assays (CCNA) also available (Table 1). Most diagnosis guidelines, including those for Europe, recommend a multiple step approach (Int1–3, 5, 7), combining EIA, NAAT, and toxigenic culture (e.g., to validate new tests) to improve diagnostic accuracy (2, 4, 16, 18–20). However, the specific combination recommended in the guidelines varies across countries and there is no clear diagnostic algorithm that applies universally (Int1–3, 5, 7). Table 1 provides additional detail based on expert consultation on which tests are conducted in case example countries, who conducts them, who pays for them and time to diagnosis.

#### 3.2.2. Challenges related to diagnosis

Diagnosing patients with CDI is challenging. There is no single test that is recommended for use alone, and the frequent use of multiple tests to arrive at a diagnosis has both time and cost implications [(2, 4, 20, 21), Int2–3, expert workshops]. In addition, laboratories within and across countries can apply diverse testing strategies due to different guidelines [(20, 22) Int2–3, 5, 7] and so there is a lack of standardized practice. There are also both advantages and disadvantages to individual diagnostic tools, related to accuracy, turnaround time and distinguishing colonization from toxigenic infection [(2, 4, 16, 20, 21, 23, 24), Int2–3].

The CDI can be underdiagnosed, overdiagnosed or misdiagnosed. Underdiagnosis can occur due to a lack of clinical suspicion, for example in younger patients or when stool does not indicate CDI, or due to diagnostic methods that are not optimal (20, 22, 25). Decisions to order CDI diagnostic tests are often influenced by patient-profile related factors (rather than symptoms alone) and the type of setting a clinician is based in (22). Clinicians in hospitals with infectious disease specialists are more likely to conduct testing for CDI than those in general hospitals, due to differences in skills and training. This can contribute to underdiagnosis (22). On the other hand, for some diagnostic tests, positive results do not always directly correlate with clinical presentation and can lead to overdiagnosis (20, 26, 27). False positive rates can also contribute to overdiagnosis (28). Performance management incentives can also have unintended consequences for overdiagnosis in light of healthcare professionals in some countries requiring permission to send samples for *C. difficile* testing (expert workshops). Some hospitals have a requirement to test for CDI in all inpatient diarrhea cases which can lead to overdiagnosis, particularly if EIA’s are used for diagnosis, due to their higher positive predictive value (Int2–3, 5). Misdiagnosis may occur when testing is performed after treatment, as *C. difficile* genetic material remain in stool weeks after infection resolves (4, 29). Complex patients, such as younger or older aged or those with co-morbidities, can also create challenges in reaching a CDI diagnosis due to difficulties in distinguishing *C. difficile* colonization from a toxigenic infection and when patients display unusual symptoms (Int2, 4, 7).

Patients can also face long waiting times for diagnosis, particularly if they present in the community, due to lack of availability of primary care physicians, physical distance from a lab, need to implement infection control measures in hospital, diarrhea being a non-specific symptom and multiple testing requirements [(2, 20, 29, 30); Int2, 4–8, expert workshops]. This can have implications for health outcomes (2, 20, 29, 30).

There are also challenges in classifying the severity of CDI, in part related to a lack of consensus on clinical markers for severity, and a reliance on clinical judgment (4, 29, 31, 32).

#### 3.2.3. Treatment of first episode CDI

Antibiotics are the main treatment used for CDI. The antibiotics used are primarily oral vancomycin, fidaxomicin and metronidazole. Vancomycin and fidaxomicin have similar efficacy (2, 4, 16, 18, 31, 33–38) and are recommended in The European Society of Clinical Microbiology and Infectious Diseases guidelines [(2, 4, 18, 33, 34, 38); Int3]. While metronidazole has traditionally been the first line treatment in the past, most countries appear to be replacing this with vancomycin and/or fidaxomicin as these have demonstrated higher efficacies for CDI. However, it is still used in some situations (2, 4, 16, 18, 19, 31–33, 35, 36, 38–42). While the choice and combination of antibiotic options vary according to national guidelines, treatment options can also vary within countries. For example, each province in Canada has its own treatment guidelines (expert workshops). The choice can also be influenced by cost considerations, e.g., fidaxomicin may not be offered as a first option in some contexts as it is more expensive (Int2–3, expert workshops).

Non-antibiotic-based treatments for initial CDI are also available for use as add-on treatments to an antibiotic regime. Surgery can be used to treat severe or fulminant CDI (2, 18, 33, 35, 40, 43). Monoclonal antibody therapy, such as bezlotoxumab, is emerging as a potential treatment that may be effective at preventing recurrences of CDI (2, 4, 18, 33, 36). Probiotics are rarely used as part of the process of treating CDI and are not recommended in guidelines given the evidence on efficacy is limited (2, 16, 29, 35, 36).

After the diagnosis of CDI, it is important for patients that any non-CDI focused antibiotic therapy or proton pump inhibitors are stopped, if possible, to prevent worsening of the infection (2, 16, 17, 31, 34, 35, 43).

Treatment decisions can be made by diverse healthcare professionals. In some countries, this is often by primary care physicians who can be the first point of contact for the patient, but for patients presenting with symptoms in hospital settings infectious disease specialists or gastroenterologists are often involved in deciding on the treatment approach (Int2, 5–7). In some countries, pharmacy staff can also be involved (Int2–3). According to interviewees across countries, patients generally have little involvement in deciding what treatment they will receive (Int3, 5–7). However, patients may have more involvement in decision-making for recurrent infections, particularly in the use of FMT (Int4–5, 7). According to one expert, the extent of patient involvement in treatment decision-making is also dependent on how receptive the clinician is to this, and how unwell the patient is (with sicker patients potentially being less involved in decision-making) (Int4).

Table 2 provides additional detail on treatment pathways, based on expert consultation, elaborating on how quickly post-diagnosis treatment commences, how treatment is initiated and by whom, whether patients are involved in treatment decision-making, the treatment course and duration and who pays for treatment in case example countries.

#### 3.2.4. Treatment challenges

Ensuring appropriate and effective treatment that is optimal for an individual patient comes with a set of challenges. For example, anti-CDI antibiotics are the first-line treatment for CDI, but can have side-effects such as a further imbalance of the gut microbiome [(2, 7, 16, 23, 33, 36, 44, 45); Int1, 3, 5–6]. Although the evidence base is inconclusive, there is also some concern about risks of resistance to mainstream therapies (18, 33, 36).

Timely treatment matters for successful outcomes, but there can be challenges to ensuring timely treatment as well. Although these appear rare (29) they are a risk, especially if diagnosis is not timely. Patients with additional complexities, such as the elderly and patients with co-morbidities, may face difficulties in treating their CDI due to frailty, multiple health issues that need addressing or a lack of response to treatment (Int2, 5).

There is a lack of evidence on the optimal treatment regime for CDI (7, 16, 33), especially for severe infections (7) and cost considerations may also play a role in what is used (as we expand on in section “3.3.3 Economic considerations”).

#### 3.2.5. Patient monitoring and follow-up

If a patient is diagnosed in the community, there is generally little follow-up across case example countries, and patients are told to return to their GP if their symptoms do not resolve (Int2, 5–7), given that in most cases infection may be mild.

Patients in hospital (either with initial or recurrent infection) are subject to closer monitoring, which primarily involves referencing stool charts, recording bowel movements, testing for white blood cell counts, assessing inflammatory marker, and, in more complex cases, CT imaging (Int2, 5, 7). This is to check for compilations such as severe dehydration, acute kidney injury, fever, ileus, and toxic megacolon (23) and side effects of medication.

Monitoring of inpatients can involve a diverse range of healthcare professionals and varies across countries. For example, in England, guidance states that effective patient care should involve weekly monitoring by a multidisciplinary team of healthcare staff, including microbiologists, infectious disease or infection prevention and control clinicians, nurses, a GI or surgeon, a pharmacist, and a dietician (31). According to interview evidence, these teams may be more frequently in place for more complex cases, such as older patients or those with underlying conditions. In Australia, ideally infectious disease specialists are primarily involved monitoring diagnosed inpatients and in Canada, data from interviews suggests specialists would not be consulted for the first CDI episode, unless it was a fulminant case which would involve gastroenterologists, infectious disease specialists and/or surgery teams (interviewee reference number withheld to preserve anonymity).

Table 2 provides additional detail on how patients are monitored and how they are assessed for cure in case example countries, based on expert consultation.

#### 3.2.6. Monitoring related challenges

Monitoring patients with CDI in hospital can be difficult as bowel movements are not always easy to record due to lack of available staff or due to a threshold of 3+ loose bowel movements over 24 h for a patient to be tested for CDI (Int5). Staff capacity constraints are the key challenge.

It can be difficult to ascertain whether a patient has been cured and whether an episode has been resolved. Some literature suggests that an initial CDI episode can be considered as ‘cured’ if symptoms resolve after 30–90 days (18), but there is a lack of consensus on this matter and toxins and genetic material from *C. difficile* can remain in the stool for several weeks after the infection is treated (16) (expert workshops).

#### 3.2.7. Managing CDI recurrence

A review by Khanna (23) states that CDI recurrence occurs in an estimated 20–30% of cases after the first CDI episodes, increasing to approximately 60% of cases after three or more episodes (23). Should CDI recurrence be suspected in a patient of any age, it is important to distinguish whether it is actual recurrence or if symptoms are due to something else, such as post-infection irritable bowel syndrome (IBS) (23). While distinguishing between recurrence and an entirely new CDI infection is also important as treatment regimes can vary, it can be difficult to achieve this in practice. Diagnosing recurrence generally involves first an assessment of symptoms and then diagnostic testing [(23, 34); Int1–3, 5, 7].

Treatment options for recurrent CDI are more diverse than for first episode infection and include therapies such as fecal microbiota transplantation (FMT), antibiotics different to those given in the initial infection such as vancomycin or fidaxomicin (if not used first time) stronger doses of antibiotics than those used for the initial episode and taper-pulse antibiotic regimes. Alternative antibiotic regimes, noted by multiple articles, are: (1) fidaxomicin; (2) taper–pulse vancomycin; (3) vancomycin or fidaxomicin followed by FMT; and (4) vancomycin followed by rifaximin (for multiple recurrences where alternatives have failed) [(2, 4, 16, 18, 29, 31, 33, 35–38, 40, 44); Int1–5, 7]. Rifaximin is recommended for patients who cannot undergo FMT (37). Metronidazole is not recommended for treatment of recurrent infections (18, 33, 35). Patients with risk factors, but FMT failure can undergo a course of antibiotics and FMT can be re-considered should recurrence occur (23). Table 3 elaborates on approaches to preventing recurrence, the diagnosis of recurrence, the treatment course and who pays for treating recurrences in case example countries, drawing on expert consultation.

#### 3.2.8. Challenges in managing recurrence

The CDI recurrences can be challenging to diagnose due to lack of monitoring for recurrence symptoms and difficulties in distinguishing recurrence from new infection [(2, 42, 45); Int2–3, 5, 7].

There are also challenges in both access to and efficacy of some treatments, for example FMT. FMT efficacy for treating recurrent CDI can be influenced by factors such as having an underlying condition [such as IBS or Irritable Bowel Disease (IBD)], the use of systemic antibiotics after FMT and being hospitalized (18, 23, 44). While FMT is generally considered safe, there are some risks of adverse events (such as abdominal discomfort, nausea, vomiting, transient diarrhea, and aspiration), infection transmission and post-infection IBS. There are also some concerns about the lack of research into long-term safety [(2, 16, 18, 23, 33, 36, 44); Int5]. Workforce capacity, facilities and resource challenges can also have an impact on access to FMT (Int2, 6).

### 3.3. Influences on patient care related to the wider healthcare system and associated challenges

Diverse features of the wider healthcare system, related to (i) access and organization of service delivery and quality of care; (ii) guidelines and regulation; (iii) economic considerations; (iv) education and awareness raising of healthcare professionals; (v) education and awareness raising for patients, and (v) COVID-19 pandemic related factors influence the care of patients with CDI. Supplementary Table 2 summarizes the key challenges applying to the case-example country contexts.

#### 3.3.1. Access and organization of service delivery and quality of care

The organization of healthcare services for patients with CDI, such as links between primary and secondary care, the set-up of outpatient care and availability of specialist CDI clinics, can influence the type of care CDI patients receive (Int3–5). The degree of multidisciplinary work may also vary, which may influence the management of some patients with CDI (expert workshops).

Access to treatments such as FMT are also a complex challenge (as introduced earlier) (36, 37). Identifying, recruiting and retaining stool donors, challenges to staff capacity and delivery facilities, lack of standardization of screening of donors, costs of testing donors and the emergence of new pathogens that need to be tested for all present access challenges [(18, 23); INT2, 4–7]. Beyond access, the lack of standardization of FMT procedures and a need for further evidence on optimal stool preparation procedures and modes of FMT delivery (e.g., colonoscopy, enema, and capsules) can also represent barriers to optimal patient care and experiences (18, 23).

#### 3.3.2. Guidelines and regulation

Guidelines for diagnosis, treatment and management of CDI can vary across countries. For example, some guidelines have different recommendations for who could be at higher risk for CDI and who should be tested for the infection, e.g., the recommended 2–3 step algorithm for testing for CDI can differ across country guidelines (43) (expert workshops).

There is also variation in guidelines on what to use as first-line treatments for CDI across countries (33), for example whether to use metronidazole. To illustrate, Canadian guidelines only recommend this in specific situations (e.g., for children, where vancomycin/fidaxomicin are not available or cannot be used) whereas Australian guidelines recommend metronidazole as a first line treatment in initial (mild) infections (31, 35, 40). Older guidelines are still more likely to recommend the use of metronidazole than more up to date ones, as well as to not include fidaxomicin as a key treatment option. However, the European Society of Clinical Microbiology and Infectious Diseases guidelines, updated in 2021, do now recommend the use of fidaxomicin as a first line treatment for initial CDI (38) but the extent to which this is reflected in individual in-country practices remains to be seen.

While guidelines on preventing and treating recurrences of CDI appear slightly more consistent across case example countries, there is still variation in the recommendations made, particularly for treating first recurrence (31, 35, 37, 40, 43). For example, 2018 Canadian and 2016 Australian guidelines recommend the use of vancomycin to treat first recurrences in adults, but 2021 English guidance recommends fidaxomicin (31, 35, 40). The Australian guidance also recommends the use of a rifaximin chaser after 2+ recurrences in adults, but this is not included in the reviewed English or Canadian guidelines (31, 35, 40). Differences in guidelines for managing recurrences may be related to factors such as new evidence emerging over time (which can be incorporated into newer guidance, but not always in a timely manner) or accumulation of evidence of one treatment being inferior (37), and wider availability and reimbursement contexts may also play a role.

While guidelines may be in place to support the treatment and management of patients with CDI in many countries, evidence suggests that these are not updated on a regular basis (Int3, 5, 7). This is a challenge to optimizing care quality. A European survey study published in 2018 noted that while national guidelines for managing patients with CDI were present in 14 (70%) of the included countries, 4 countries had not revised the guidelines within the last 5 years (34).

Guidelines are also often modified or applied inconsistently in clinical practice (7, 19, 30, 39). There may be good reasons for doing so, but this merits further research. For example, Turner et al. (30) note that there can be a risk of clinicians prescribing treatment for CDI based on a positive result from a single test, as opposed to the recommended 2–3-step algorithm of multiple tests (30). A lack of adherence to guidelines may also be in part affected by a lack of auditing practices on adherence (34) or due to a lack of a local policies and protocols on CDI treatment (39). Although meriting further research, not all clinicians necessarily read updated CDI guidelines in detail due to their length (expert workshops).

#### 3.3.3. Economic considerations

Several financial resource related considerations can have an impact on the care of patients with CDI and give rise to challenges. The cost of some anti-CDI antibiotics may be difficult for healthcare systems to absorb (2, 33) and this may also be a challenge in relation to emerging treatments, e.g., monoclonal antibody therapy (workshops). Fidaxomicin is more costly (in terms of acquisition) compared to metronidazole and vancomycin, which may influence its availability in some settings (34, 36, 40, 45), despite some emerging evidence suggesting that fidaxomicin is more cost-effective than other antibiotics for both initial and recurrent CDI in most situations due to the reduced risk of recurrence, despite higher upfront cost (46).

While data on the cost of recurrence is more limited compared to initial infection, evidence from two articles suggests that recurrent CDI costs more to treat than initial infection, likely due to higher severity and longer lengths of hospital stay (46, 47). For example, a 2018 study using data from 45 patients from the UK indicated that length of stay for patients with recurrent infection was 33 days, significantly longer than the 17 days for those with initial infections (47). Intensive care unit stays were also found to be longer for patients with recurrent infections compared to initial infections in this study (2.5 vs. 0.7 days, respectively) (47). Treatment, pathology tests, sterile services, linen, medical pay and overheads have also been found to cost more in recurrent CDI compared to initial infection (47). FMT is generally considered to be cost-effective for treating recurrent CDI (46, 48), but there is a need for further research on how wider healthcare systems factors such as setting up and maintaining stool banks may impact on cost effectiveness.

Cost can be a barrier not only to optimizing treatment, but also to optimal diagnostic test use and may contributes to some of the variation seen in CDI guidelines across countries (Int2–3, 5–6, expert workshops).

In some countries (e.g., Canada), reimbursement for treatment varies across provinces which impacts on efforts to standardize practices at a national level and results in subsequent variation in treatment regimens (expert workshops).

Litigation costs and hospitalization costs can also present financial challenges (expert workshops) (49).

#### 3.3.4. Education and awareness raising for patients and clinicians

Patient-related issues such as stigma, disgust and embarrassment or low awareness and understanding of CDI symptoms can be a barrier to timely diagnosis. This can lead to patients delaying seeking help from a healthcare professional or not providing all the information about their symptoms (e.g., appearance of bowel movements) (Int2, 4, 6, 8, expert workshops). A scarcity of public health campaigns (national and regional) about CDI symptoms and the importance of seeking care can also impact on access to the right care at the right time and place (expert workshops). It can also impact on resorting to treatments for which sufficient evidence may be lacking, such as probiotics (expert workshops).

There is limited evidence on the impact of CDI from the patient or care-giver perspectives, and this is an area that requires further research. One Canadian study explored the impact on patient’s quality of life (QoL) as a result of CDI by conducting a survey of 167 people with CDI and their carers (29). QoL was ranked from 1 (patient is unable to care for self and requires hospital care) to 6 (patient can undertake normal day-to-day activities without support). The results indicate that those patients who report a lower QoL before CDI experience a larger impact on their QoL when they have the infection. Moreover, carers reported that patients had lower QoL scores than the patients reported about themselves (median QoL of 3 compared to 4, respectively).

Clinician awareness and knowledge of *C. difficile* diagnostic, treatment and referral processes can also be relatively low, especially in primary care and amongst some specialist clinicians such as surgeons across the case example countries (expert workshops). This is partly due to CDI not having prominence in the medical curriculum (or having not been there in the past) and lack of awareness of guideline updates (Int3, 5–8, expert workshops). The extent to which healthcare providers discuss bowel movements with patients in a way conducive to identifying a potential case of CDI can also influence whether or not a patient is tested for CDI (Int4, 6, expert workshops). There can also be risks from clinicians not interpreting test results correctly and treating a patient in cases where *C. difficile* has been detected but is not toxigenic (expert workshops).

#### 3.3.5. Impact of COVID-19 on the CDI care pathway

Unforeseen events such as the COVID-19 pandemic can have an impact on access to care and care quality. In some contexts (e.g., the UK), data suggests that cases of CDI (particularly hospital acquired infection) rose during the pandemic and the 30-day case fatality rate for CDI also increased, and this has been associated with COVID-19 (8, 50). Although evidence of the impact of COVID-19 on the care of patients with CDI is currently scarce and inconclusive, consultations with experts noted that in some case example countries, the prioritization of dealing with COVID-19 increased risks related to timely diagnosis and treatment of patients with CDI symptoms (expert workshops, Int4–6) and that patients may have avoided seeking healthcare due to fears of contracting COVID-19 (Int8). In the UK and France FMT services were stopped by regulatory agencies during the early stages of the pandemic.

### 3.4. Priorities for improving the care of patients with CDI

Based on insights into the CDI care pathway and associated challenges outlined previously and as informed by the narrative review and stakeholder consultations, a survey was developed to explore priority areas where taking action could help improve patient care. Respondents across case example countries were asked to select which improvement opportunities they thought were most important within the following categories derived through thematic analysis of literature, interview and workshop data: (1) diagnosis and treatment, (2) access and organization of service delivery and quality of care, (3) guidelines and regulations, (4) education and awareness raising for clinicians, (5) education and awareness raising for patients. (In addition to selecting the most important opportunities-i.e., top priorities, respondents also rated opportunities. To avoid repetitiveness and in light of consistent messaging, our analysis focuses on the selection of the most important opportunities rather than rating data). The survey also explored evidence gaps that need addressing. Respondents were asked to select the top third threshold in terms of importance, amongst a list of actions in each thematic area (so that in a list of ten items, for example, they were able to select up to three, if there was a list of six, they could select up to two). Throughout, where presenting the findings, we highlight the actions where 50% or more respondents reported it as a priority area for improvement (as a threshold for strong agreement), but we also reflect on the wider sentiment across survey respondents (i.e., commenting on areas where 30% of more of survey respondents felt an action was a priority).

#### 3.4.1. Diagnosis and treatment

**FIGURE 1:**
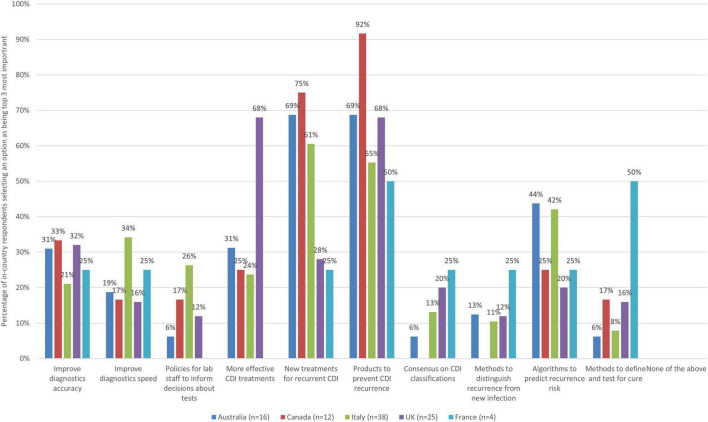
The improvement actions related to diagnosis and treatment of CDI that are the most important (bars represent the percentage of respondents that rated each improvement action as one of the most important by country).

Amongst a list of ten improvement actions related to diagnosis and treatment, there was strong agreement across all surveyed countries that innovative products for preventing recurrence of CDI was a key priority (see Figure 1). This option was selected by 92% of respondents from Canada, 69% from Australia, 68% from the UK, 55% from Italy, and 50% from France, although due to low response numbers, this equated only two respondents for France.

Developing innovative, more effective treatments for treating recurrent CDI was also seen as a key priority by respondents in three countries (75% Canada, 69% Australia, and 61% Italy). Although this option was seen as a top priority by some respondents from the UK and France as well (28% of UK and 25% of respondents from France), the relative strength of sentiment about its importance was lower across respondents in these countries.

When looking at country-level insights, some key findings are captured in Table 4.

**TABLE 4:** Country-level insights relating to diagnosis and treatment priorities.

Country	Insights
Australia	• Strong agreement that developing more effective treatments for treating recurrent CDI and developing innovative products for preventing recurrent of CDI were top priorities (69% of respondents for both), mirroring findings from the survey overall. • Some other areas were also seen to be a priority, but by fewer than half of all respondents. For example, 44% of respondents from Australia saw developing algorithms to more accurately predict the risk of CDI recurrences as a top priority. Just under a third (31%) saw improving the accuracy of diagnostic methods and developing more effective treatments for recurrent CDI as top priorities. • Other options were selected more rarely (less than 30% of respondents).
Canada	• Two options stood out as key priorities (aligned with overall survey findings) with very strong agreement across survey respondents. Developing products to prevent recurrence was seen as a top priority by 92% of respondents. Three quarters (75%) saw developing new treatments for recurrent CDI as a key priority. • Improving diagnostic accuracy was seen as less of a priority, although it was still selected by one-third (33%) of respondents. • Other improvement areas were selected as priorities more rarely (less than 30% of respondents). • No respondents from Canada felt that there is a need for consensus related to classifications of CDI nor for improved methods to distinguish recurrence from new infection (unlike in other countries).
Italy	• Over half of respondents saw developing new treatments for recurrent CDI (61%) and products to prevent recurrence (55%) as key indicating strong agreement on these priorities (in agreement with views from Australia and Canada). • However, there was a variety of views, with all other improvement actions also seen as a top priority by at least some respondents and to varying degrees. For example, 42% saw developing algorithms to predict risk of recurrence as a top priority and 34% felt improving the speed of diagnosis is a key priority. • Other options were selected as priority more rarely (less than 30% of respondents).
UK	• There was strong agreement on the importance of two improvement areas (68% of UK respondents selected them as a top priority), these being developing more effective treatments for treating initial CDI and develop innovative products for preventing recurrence of CDI. • Interestingly, unlike in Australia, Canada and Italy, developing new treatments for recurrent CDI did not emerge as a top priority in the UK (selected by 28% of respondents). • Significantly fewer respondents saw other options as a top priority. While improving the accuracy of diagnostic methods was seen as a top priority by 32% of respondents, no other option was seen as a priority by 30% more of the UK respondents.
France	• There is no strong agreement on what the top improvement priorities within the diagnosis and treatment space are (this may partly relate to a low number of survey responses). • Six actions made it into the top third threshold in terms of priority actions. • Methods to define and test for cure was selected as a key priority by 50% (two out of the four) respondents from France.

#### 3.4.2. Access and organization of service delivery and quality of care

**FIGURE 2:**
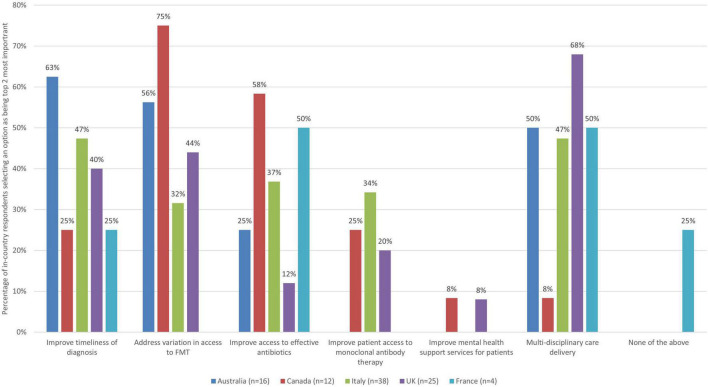
The improvement actions related to access and organization of service delivery and quality of care for CDI that are the most important (bars represent the percentage of respondents that rated each improvement action as one of the most important by country).

As there were six improvement actions to select from in the theme of access and organization of service delivery and quality of care, respondents were asked to select up to two (top third threshold) improvement actions they thought were the most important, and thus represent priorities (Figure 2).

When looking across countries, there are both some similarities, but also notable differences and a variety of views on key areas in need of improvement as they relate to this theme. For example, addressing variation in access to FMT at local, regional and national levels was seen as a priority area for improvement by over half of all respondents from Canada and Australia (75 and 56%, respectively), but this was not the case for the UK (44%), Italy (32%), or France (0%). Half or more of respondents from Australia (63%) and France (50% – although this is only 2 respondents) saw improving timeliness of diagnosis as a top priority, and Italy was close (47%), but this was not the case for the UK (40%), or Canada (25%). Improving access to effective antibiotics for treating CDI was seen as a top priority for over half of respondents from Canada and France (58 and 50%, respectively), but this was not the case for Italy (37%), Australia (25%), or the UK (12%). Facilitating more multi-disciplinary care delivery in the management of patients with CDI was selected within the top improvement actions by half or more of respondents from the UK, Australia, and France (68, 50, and 50%), nearly half in Italy (47%), but much less in Canada (8%).

When looking at country level data, there are both similarities and differences in views about improvement priorities related to access and organization of services and quality of care. Although speculative and meriting further research, these may have to do with some differences in the way healthcare systems are organized in terms of provision of care to patients with CDI and unique challenges. See Table 5.

**TABLE 5:** Country-level insights relating to access and organization of service delivery, and quality of care priorities.

Country	Insights
Australia	• Half or more of survey respondents saw improving timeliness of diagnosis (63%), addressing variation in access to FMT (56%) and improving multi-disciplinarity of care delivery (50%) as top priorities – signaling strong agreement on these issues. • No other option had more than 30% of survey respondents seeing it as a top priority. • No respondents from Australia saw improving mental health support for CDI patients as a priority.
Canada	• Addressing variation in access to FMT and improving access to effective antibiotics were seen as key proprieties (selected by 75 and 58% of respondents, respectively). • No other option received a top priority status from 30% or more of respondents.
Italy	• The same proportion of respondents selected two actions as most important (47%): improve timeliness of diagnosis and facilitate more multi-disciplinary care delivery. • However, there was no strong agreement as no single area was chosen as a top priority by over half of the respondents and this was an illustration of a heterogeneity of views. • Other options selected as a priority by fewer respondents included improving patient access to antibiotics (37%) and to monoclonal antibody therapy (34%) and addressing variation in access to FMT (32%). • No respondents from Italy saw improving mental health support for CDI patients as a priority.
UK	• There was a strong agreement on improving multi-disciplinary care delivery as a top priority (68%) but a variety of views on other priorities, with 44% of respondents seeing addressing variation in FMT as top priority and 40% seeing improving the timeliness of diagnosis as top priority. • No other option had 30% or more of respondents selecting it as a top priority.
France	• The same proportion of respondents selected two actions as most important (50% for both, although this is only 2 respondents): improving access to effective antibiotics and more multi-disciplinary care delivery. • In addition, 25% of respondents (1 respondent) selected improved timeliness of diagnosis as most important. One felt that none of the listed actions were priorities.

#### 3.4.3. Guidelines and regulations

**FIGURE 3:**
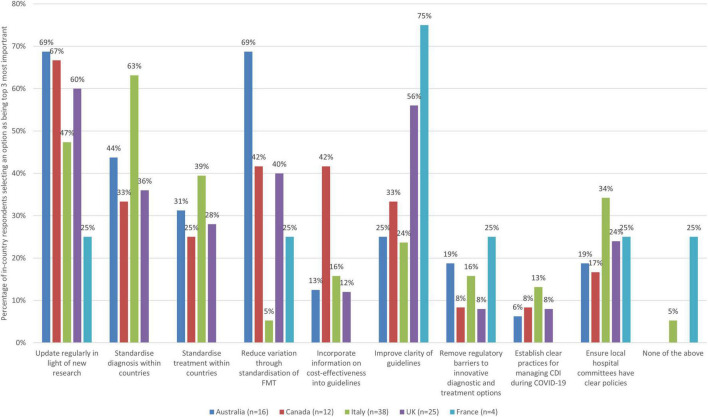
The improvement actions related to guidelines and regulation for CDI treatments that are the most important (bars represent the percentage of respondents that rated each improvement action as one of the most important by country).

Amongst a list of nine improvement actions related to the theme of guidelines and regulation, respondents were then asked to select up to three (top third) they saw as most important (Figure 3).

When looking across the participating countries, it is notable that more than half of respondents from most surveyed countries saw updating diagnosis and treatment guidelines more regularly as a top priority (69% for Australia, 67% for Canada, and 60% for the UK) and nearly half for Italy (47%), but this was not the case for France (25%, 1 respondent). More than half of respondents from Italy (63%) also felt that standardizing diagnosis guidelines within countries is of key importance, but this was not the case for other surveyed countries (44% Australia, 36% UK, 33% Canada, and 0% France). More than 50% of respondents from Australia (69%) felt that guidelines to help with standardization of FMT practice would be very important as well, but this was not the case for other countries (42% Canada, 40% UK, 25% France, and 5% Italy). Over half of respondents from France and the UK (75 and 56%) respectively felt that improving guideline clarity was a priority, but significantly fewer from Canada, Australia and Italy selected this as a top priority (33, 25, and 24%, respectively).

Considering country level data, some notable findings are presented in Table 6.

**TABLE 6:** Country-level insights relating to guideline and regulation priorities.

Country	Insights
Australia	• Strong agreement on the importance of updating diagnosis and treatment guidelines more regularly in light of new research and also saw guidelines to reduce unwarranted variation through standardization of FMT practice as a priority (69%). • However, there was a variety of views on priority areas, with 44% of respondents also noting standardizing diagnosis practices within the country as a top priority and standardizing treatment guidelines within countries was seen as a priority by 31% of respondents. • Other improvement options were seen as important by fewer than 30% of respondents.
Canada	• Strong agreement on only one option – updating diagnosis and treatment guidelines regularly in light of new research (67% selected this as a top priority). • There was a variety of views on other priority areas, with 42% seeing reducing unwarranted variation through standardization of FMT practice and incorporating information on cost effectiveness into guidelines as most important areas where improvement is needed. 33% saw standardizing diagnosis guidelines within countries and improving clarity of guidelines as most important. • Other improvement options were seen as important by less than 30% of respondents.
Italy	• Strong agreement about the importance of standardizing diagnosis guidelines within countries (63% of survey respondents selected this to be an important option). • However, there is a variety of views on the importance of other improvement opportunities. For example, 47% of respondents from Italy saw updating guidelines regularly as a top priority, 39% saw standardizing treatment guidelines as key and 34% saw ensuring local hospital committees have clear policies as most important. Other options were seen as important by less 30% of participants.
UK	• Strong agreement about the importance of updating guidelines in light of new research (60% of respondents) and improving clarity of guidelines (56%). • No other area was seen as a top priority by half or more of survey respondents, but 40% saw reducing unwarranted variation through standardization of FMT practice as a top priority. Less frequently selected as key was standardizing diagnostic guidelines (36%). • Other improvement options were seen as a top priority by less than 30% of respondents, but do illustrate the heterogeneity of views.
France	• Three-quarters of respondents selected improving clarity of guidelines as a top priority. • Other improvement options were seen as a top priority by less than 30% of respondents and no respondents from France selected the other options.

#### 3.4.4. Education and awareness raising for patients

**FIGURE 4:**
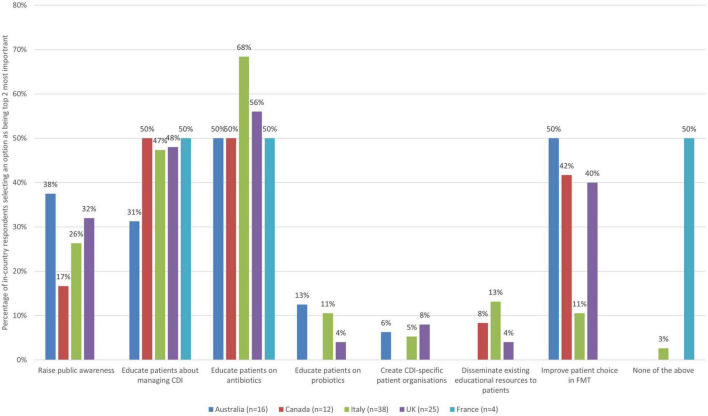
The 2 improvement actions related to education and awareness raising for patients that are the most important (bars represent the percentage of respondents that rated each improvement action as one of the most important by country).

As there were seven improvement actions to select from in the theme of education and awareness raising for patients, respondents were asked to select up to two (top third) improvement actions they thought were the most important (Figure 4).

When looking at the responses across countries, a number of similarities emerge, but also some unique perspectives. For example, half or more respondents from all countries saw educating patients with CDI on the appropriate use of antibiotics as a top improvement action (68% Italy, 56% UK, and 50% for Australia, Canada, and France). Half of respondents from France and Canada, and nearly half of respondents from the UK (48%) and Italy (47%) also felt that educating patients with CDI about the management of the illness and the potential future impact on their lives was a priority area for improvement, but this was not the case for Australia (31%). Improving patient choice in relation to FMT was selected as a priority action by 50% of respondents from Australia, but by fewer than half of respondents from other countries (42% Canada, 40% UK, 11% Italy, and 0% France).

When zooming into country level data, some notable findings are presented in Table 7.

**TABLE 7:** Country-level insights relating to education and awareness raising for patients priorities.

Country	Insights
Australia	• Half of respondents saw educating patients with CDI on the appropriate use of antibiotics and improving patient choice in relation to FMT as priority actions. • In addition, 38% selected raising awareness of CDI among the public and 31% educating patients on management of CDI and potential future impacts as most important. • All other actions were seen as most important by fewer than 30%, and none selected improving dissemination of existing educational resources to patients as a top priority.
Canada	• Half of survey respondents saw two improvement areas as standing out in terms of importance, the one being educating patients with CDI on the appropriate use of antibiotics and the other educating patients with CDI about the management of the illness. • In addition, 42% saw improving patient choice in relation to FMT as a priority action. • All other actions were selected as most important by fewer than 30% of respondents from Canada. None selected educating patients on probiotics or creating CDI-specific patient organizations as top priorities.
Italy	• For Italy, more than half of respondents selected educating patients on appropriate use of antibiotics as the top priority (68%). • Nearly half (47%) selected educating patients with CDI about the management of the illness as a top priority. • All other actions were selected as most important by fewer than 30% of respondents.
UK	• More than half of respondents saw the need for educating patients on appropriate use antibiotics as the top priority (56%). • Some other priorities, though not with strong agreement across respondents were educating patients with CDI about the management of the illness (48%), improving patient choice in relation to FMT (40%) and raising awareness of CDI among the public (32%) as top priorities. • All other actions were selected as most important by fewer than 30%.
France	• The same proportion of respondents saw two actions as most important (50% for both, 2 respondents): educating patients with CDI on the appropriate use of antibiotics and educating patients with CDI about the management of the illness. • Half of respondents selected ‘none of the above.’ • No respondents from France selected that any of the other improvement actions were most important.

#### 3.4.5. Education and awareness raising for clinicians

**FIGURE 5:**
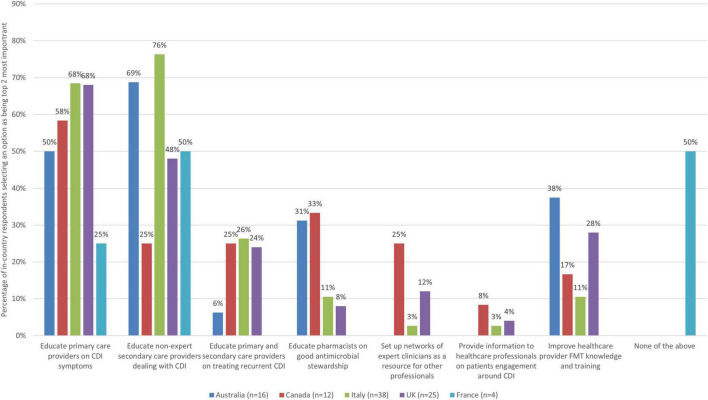
The 2 improvement actions related to education and awareness raising for clinicians that are the most important (bars represent the percentage of respondents that rated each improvement action as one of the most important by country).

As there were seven improvement actions to select from in the theme of education and awareness raising of clinicians, respondents were asked to select up to two (top third threshold) improvement actions they thought were the most important (Figure 5).

There was clear agreement on the top two priority actions. In most countries, educating and supporting healthcare professionals in primary care was seen a top priority improvement action (68% of respondents from Italy, 68% from UK, 58% from Canada, and 50% from Australia), but this was not the case for France (25%, 1 respondent). Half or more of respondents from Italy (76%), Australia (69%) and France (50%, 2 respondents), and nearly half of respondents from the UK (48%) saw educating and supporting healthcare professionals in secondary care who are not experts regularly dealing with patients with CDI as a top priority, but this was not the case for Canada (25% selected as most important).

When zooming into country level data, some notable findings are indicating relatively strong alignment between views from different countries (Table 8).

**TABLE 8:** Country-level insights relating to education and awareness raising of clinician’s priorities.

Country	Insights
Australia	• There was clear consensus on the top two priorities, selected by at least half of respondents. These were educating non-expert secondary care providers on dealing with CDI (69% selected as most important) and educating and supporting healthcare professionals in primary care (50%). • In addition, 38% saw improving healthcare provider FMT knowledge and training as most important, and 31% saw educating pharmacists on good antimicrobial stewardship as most important. • All other actions were selected by fewer than 30% of respondents, and no respondents from Australia saw setting up networks of experts providing information and educational support for healthcare professionals on how to effectively and confidently engage with patients as priority actions.
Canada	• Only one action was selected as most important by more than half of respondents: educating and supporting healthcare professionals in primary care (58%). • In addition, 33% saw educating pharmacists on good antimicrobial stewardship as most important. • All other actions were selected by fewer than 30% of respondents.
Italy	• There was clear consensus on the top two priorities, selected by over half of respondents. These were educating non-expert secondary care providers on dealing with CDI (76%) and educating and supporting healthcare professionals in primary care (68%). • All other actions were selected as most important by fewer than 30% of respondents.
UK	• Over half of respondents (68%) saw educating and supporting healthcare professionals in primary care as most important. • This was followed by 48% selecting educating non-expert secondary care providers on dealing with CDI as most important. • All other actions were selected as most important by fewer than 30%.
France	• For France, half of respondents (2 respondents) selected educating non-expert secondary care providers on dealing with CDI as most important. • In addition, 50% also selected ‘none of the above.’ • All other actions were selected as most important by fewer than 30%, with no respondents selecting four of the options as important.

#### 3.4.6. Evidence gaps

**FIGURE 6:**
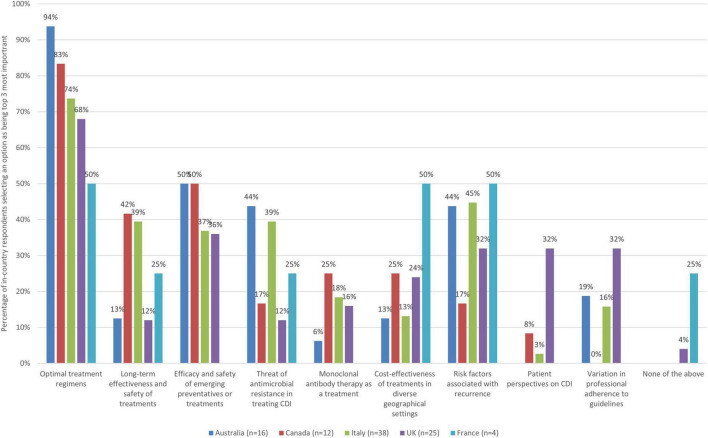
The 3 evidence gaps in relation to CDI that are the most important to address by country.

Respondents were asked to select areas where there are particularly important gaps in evidence that need to be addressed to support evidence-based practice and high-quality care for CDI. As there were ten evidence gaps to select from, respondents were asked to select up to three (top third threshold) improvement actions they thought were the most important (Figure 6).

Better evidence on optimal treatment regimens in managing patients with specific profiles was the most selected evidence gap across all countries (94% Australia, 83% Canada, 74% Italy, 68% UK, and 50% France saw it as a top priority). Half of respondents from Australia and Canada saw a need to address gaps and improve evidence on the efficacy and safety of emerging preventatives as a priority, but this was not the case for the Italy (37%), UK (36%), or France (0%). Half of respondents from France, and nearly half from Italy (45%) also identified a need for better evidence on risk factors associated with recurrence as a top evidence gap to address whereas this was not the case for other countries (44% Australia, 32% UK, and 17% Canada).

When zooming into country level data, some notable findings are presented in Table 9.

**TABLE 9:** Country-level insights relating to evidence gaps priorities.

Country	Insights
Australia	• One evidence gap was selected by nearly all respondents (94%): better evidence on optimal treatment regimens in managing patients with specific profiles. • There was also strong agreement on another: 50% saw better evidence on efficacy and safety of emerging CDI preventatives/treatments as most important to address. • In addition, 44% of respondents from Australia thought both better evidence on the threat of AMR in the treatment of CDI and better evidence on risk factors associated with recurrence were most important. • All other evidence gaps were selected by fewer than 30% of respondents. No respondents from Australia saw better evidence on the patient perspective of CDI as most important to address.
Canada	• The majority of respondents (83%) saw better evidence on optimal treatment regimens in managing patients with specific profiles as the most important evidence gap. • Half saw better evidence on efficacy and safety of emerging CDI preventatives/treatments as most important and 42% saw better evidence on long-term effectiveness/safety of treatments key to address. • All other evidence gaps were selected by fewer than 30% of respondents. No respondents from Canada saw better evidence on variation in professional adherence to guidelines as most important to address.
Italy	• Nearly three-quarters of respondents (74%) felt that better evidence on optimal treatment regimens in managing patients with specific profiles was the most important evidence gap to tackle. • In addition, 45% selected better evidence on risk factors associated with recurrence and 39% selected both better evidence on long-term effectiveness/safety of treatments and better evidence on the threat of AMR as most important. • All other evidence gaps were selected by fewer than 30% of respondents.
UK	• 68% of respondents better evidence on optimal treatment regimens in managing patients with specific profiles as the most important evidence gap. • In addition, 36% selected better evidence on efficacy and safety of emerging CDI preventatives/treatments as most important. Nearly one-third (32%) selected three evidence gaps as most important: better evidence on risk factors associated with recurrence, on the patient perspective of CDI and on variation in professional adherence to guidelines. • All other evidence gaps were selected by fewer than 30% of respondents.
France	• The same proportion of respondents selected three gaps as most important (50% for all, however, this only represents 2 respondents): better evidence on optimal treatment regimens in managing patients with specific profiles, on the cost-effectiveness of treatments in diverse geographical settings and risk factors associated with recurrence. • All other evidence gaps were selected by fewer than 30% of respondents. No respondents from France selected better evidence on efficacy and safety of emerging CDI preventatives/treatments, on monoclonal antibody therapy as a CDI treatment, patient perspective on CDI or variation in professional adherence to guidelines as most important.

#### 3.4.7. Other improvement opportunities and evidence gaps

In the survey, respondents were also provided the opportunity to share views on any additional improvement activities and evidence gaps that had not been included in the survey already. For improvement opportunities, many responses focused on infection prevention and control (which is out of scope for this study). Some stressed evidence gaps or improvement actions that had already been included in the survey questions. Additional improvement actions that were identified included: improving diagnosis of *C. difficile* carriers where the patient has active chronic inflammation of the bowel (e.g., IBD), improving the exclusion of other (non-CDI) causes of diarrhea (e.g., other infections and laxatives) to improve antibiotic stewardship, improving methods for collecting data on stool frequency and consistency, and general improvements to antibiotic prescribing.

Respondents also shared views on some additional evidence gaps related to infection prevention and control or reinforced evidence gaps that had already been covered in the survey. Additional evidence gaps that were mentioned included: better evidence on particular treatment regimes (e.g., for the first CDI episode to prevent recurrences), co-managing CDI and IBD, making a reliable diagnosis (e.g., interpreting test results and identifying cure), documenting the biological mechanisms of CDI, providing treatment in cases of positive test results but no clinical symptoms, involvement of community pharmacists alongside primary care, FMT (e.g., for first episode CDI and using synthetic material), and research into the prevention of recurrences.

## 4. Discussion

### 4.1. Reflecting on improvement priorities and future research needs

This paper contributes to understanding key challenges and areas of need of improvement in the care of patients with CDI, as they relate to the clinical care pathway and the wider healthcare system which frames its operations. In doing so it contributes to the knowledge base on how patient care could be optimized, considering similarities and differences in a sample of high-income country contexts (i.e., case example countries), and in light of the wider literature that covers a broader set of geographies and contexts.

When examining the findings, it is striking that there are both similarities and differences in priority areas for improvement in different contexts. However, what is recognized across the different examined geographies is the need for improvement actions targeting *both* innovation for clinical care directly (e.g., developing innovative treatments) and those targeting the way healthcare systems enable high quality care (e.g., through keeping guidelines up to date, education and awareness raising efforts, and health system organization).

In the following, we discuss lessons learned from the stakeholder survey and how they relate to broader ideas about challenges from the literature, expert interviews and workshops.

In doing so we focus on areas of agreement but recognize that there are also improvement actions where there was less consensus, but which are still important to segments of the populations involved in patient care.

When considering survey insights on improvement needs related to the clinical care pathway, we observed high levels of agreement on the need to develop innovative products for *preventing* recurrence across all surveyed countries and, in most case example countries, developing innovative and more effective *treatments* for recurrent CDI was also seen as a priority (Italy, Australia, Canada and, for prevention only, the UK). This resonates with insights from the literature (as reported on earlier in this paper) flagging higher demands on healthcare services in terms of managing and dealing with recurrence. In addition, in the UK there was strong agreement on the need for developing new treatments for initial CDI. This may be due to the UK having had performance management in place for decades for the management of CDI (e.g., targets and objectives) and so experts may be sensitized to the need to improve treatment options. In light of wider treatment challenges for initial CDI identified in the analyzed literature and stakeholder consultation, further research is needed to understand whether key improvements are needed in clinical effectiveness, cost-effectiveness or reduction of side-effects.

When exploring insights related to patient monitoring, the reviewed literature and stakeholder consultation identified challenges with knowing when a patient is cured, which could have implications on patient treatment decisions and healthcare resource utilization. This did not come up as an area of priority in terms of improvement in the survey data, but evidence suggests a need for further research on how to assess ‘cure’ (for example how long symptoms need to be absent before a patient is considered cured and how to accurately test if a patient is cured).

In terms of access and organization of service delivery and quality of care, areas where agreement on the need to improve was the strongest included actions to address variation in access to FMT at local, regional and national levels (Canada and Australia) and actions to facilitate more multi-disciplinary patient care (UK, Australia, and France). Improving access to FMT also resonates with findings from the conducted literature review, workshops and interviews. In addition, respondents from some countries identified improvement priorities related to access to effective antibiotics for treating CDI (Canada and France) and timeliness of diagnosis (Australia and France). Views on priorities in terms of improving organization of service delivery and quality of care were particularly diverse within Italy where there was no strong agreement on any one area being most important, but with five areas being seen as priorities by a third or more of survey respondents: improving timeliness of diagnosis, facilitating more multi-disciplinary care delivery, improving patient access to antibiotics, improving access to monoclonal antibody therapy and addressing variation in access to FMT. In general, the observed similarities and variety across surveyed countries is likely to derive from specificities of healthcare system organization, capacity and infrastructure.

Our research also points to the impact of guidelines and regulation on care quality, and to the scope to improve guideline contents and the wider support that healthcare systems can provide to improve adherence. There was strong agreement amongst respondents from the majority of surveyed countries on the importance of updating diagnosis and treatment guidelines in light of new knowledge, with over half of respondents in Australia, Canada, and UK seeing this as a top priority, and nearly half in Italy. This resonates with insights obtained through international interviews and literature on European practices, which highlight outdated guidelines being a challenge to optimizing patient care. Although warranting further research, outdated guidelines may also be linked to challenges in adherence to guidelines identified in the literature and discussed earlier. Here, it is important to note that The European Society of Clinical Microbiology and Infectious Diseases guidelines for treating CDI were updated in late 2021, but progress with the implementation of the new guidelines remains to be seen. Other factors such as economic resource constraints were also identified in the literature and in stakeholder consultation as impacting on the feasibility of adhering to some guidelines. Clinician preferences and lack of audit– as discussed in the literature and reported on earlier- may also play a role in guideline adherence. In Italy, but not other surveyed countries, there was strong agreement that standardizing diagnosis guidelines was important, perhaps due to diverse practices in terms of diagnostic testing in different parts of the country. In Australia standardization was seen as important in the context of FMT practice in particular. In the UK and France, improving guideline clarity was also seen as a priority by half or more of respondents.

Our research flagged that engaging with patients with CDI around education and awareness raising on the appropriate use of antibiotics is also important for healthcare systems to consider as part of efforts to improve patient outcomes. There was strong agreement on this across respondents in all surveyed countries. Other related priority actions where there was strong agreement amongst respondents within some countries included educating patients about the management of CDI and the potential impact of the disease on their lives, (Canada and France) and improving patient choice with respect to FMT (Australia). Importantly, the survey targeted clinical and scientific experts, and did not flag combating stigma or embarrassment as a key priority, but this is a challenge identified in other stakeholder consultation (e.g., interviews and workshops), particularly from patient representatives, and merits future consideration.

Finally, information and knowledge gaps were also identified as an area for attention in terms of future actions within healthcare systems. In most case example countries (Italy, UK, Canada, and Australia) survey respondents saw as top priority the need to educate and support primary care professionals on identifying CDI symptoms, when and how to test and diagnose patients with CDI (or refer for testing and treatment to a specialist) and how to manage patients who are being treated. Educating and supporting healthcare professionals in secondary care who are not experts regularly dealing with patients with CDI was also identified as needing attention and being a priority in some countries (Italy, Australia, and France). This resonates with the challenges identified in the analysis of the literature and stakeholder consultation, especially in the context of risks of underdiagnosis and misdiagnosis and potential challenges associated with time to diagnosis, as discussed earlier in this paper.

Our analysis also identified diverse evidence gaps which would need to be addressed to support optimal patient care. In reflecting on the insights gained, it is clear that tackling any future research agenda calls for both basic science, social science and health systems research approaches and perspectives, as both clinical and behavioral evidence gaps exist in the current knowledge base. Ambitions to improve patient care will therefore depend on the ability to orchestrate clinical practice interventions and wider behavioral and systems-level actions. It would also be important to evaluate the impact of any interventions over time, both in terms of impacts on patient health and quality of life, but also on wider society and any economic implications.

Reflecting on insights from the stakeholder survey, the need for further research on optimal treatment regimens for patients with different profiles stood out as an area where there was strong agreement on this being a priority for a future research agenda. Given the survey respondents largely represent clinical experts, this is not surprising, but it also resonates with findings from the literature review, particularly in the context of challenges with treating patients who may be elderly, frail, with complex needs or comorbidities. Research into optimal treatment regimens would need to consider both clinical and cost-effectiveness, and patient experience. In some countries, survey respondents also placed particular emphasis on improving the evidence base on preventing recurrence – such as evidence on the safety and efficacy of emerging preventatives/treatments (Australia, Canada), and in France (though only a small absolute number of respondents), better evidence on risk factors associated with recurrence and better evidence on treatment cost-effectiveness were also seen as key areas meriting more research.

However, when reflecting on the overall insights gained from the literature, interviews and workshops, it is clear that improving patient care calls for advances in research in a number of other areas as well. For example, the analysis and triangulation of the stakeholder consultation data from multiple sources (e.g., interviews, workshops, and survey) and literature suggests needs to also conduct additional research on how CDI affects patient quality of life and also the experience of carers; on how potential stigma and disgust in discussing bowel problems impacts on patients accessing care, and research into the nature of interactions between patients and healthcare professionals.

We also explored variations in practice, and these too point to avenues of relevance for a future research agenda. For example, we noted diversity in referral behaviors both within and across countries (e.g., whether a patient who presents to a community care setting is referred to gastroenterologists or infectious disease specialists, or elsewhere in the system); diversity as to where diagnostic testing takes place (e.g., in public or private labs); in the combination and order of use for diverse tests used to diagnose patients, in the choice and combination of antibiotic options used to treat patients, and in the degree of multidisciplinary care involved in monitoring patients. Some of this variation in practice may be warranted in light of patient symptoms and healthcare system organization, while other areas of variation may be more subject to personal preferences and experiences of healthcare professionals or resource and capacity constraints. Further research is needed to explore where variation may or may not be warranted. For example, our evidence suggests that the frequent use of multiple diagnostic tests has both time and cost implications and there may be scope to optimize practices through further research on optimal diagnostic algorithms for patients with different profiles (given that the use of diverse algorithms was identified as a challenge).

Finally, whereas this research is unique in adopting a multidisciplinary, clinical practice and health services research perspective on the care of patients with CDI, and in combining a narrative review covering diverse high income country contexts with in-depth case examples of five countries, further primary research is needed to complement the findings identified through the case examples with data from other countries. We hope the insights we have shared in this paper help inform future research agendas, as well as shed new light on the diverse and complementary ways in which the care of patients with *C. difficile* infection can be improved in the future.

### 4.2. Limitations

This study examines the CDI patient care pathway and discusses key challenges to optimizing patient care across the pathway- from diagnosis, to initial treatment, patient monitoring and management of recurrence. It also examines key priorities in the context of areas where improvement is needed and explores variation in views across and within countries. It is also novel in that is covers multi-disciplinary factors, bringing together clinical and healthcare service and systems perspectives and drawing on diverse evidence sources – narrative review, interviews, workshops and a survey.

There are, however, some limitations to note. Firstly, the narrative review did not include all possible articles on the topic of the CDI patient pathway. It was intended to be a focused review of key relevant evidence to identify key challenges and improvement opportunities, not a full systematic review, but following many core principles of a systematic review approach. While the consultations engaged key experts in the five country examples, a limited number of individuals were consulted through interviews (eight in total), but this was mitigated with wider survey-based consultation. Variation in clinical practice and service delivery across provinces and states of larger countries (e.g., Australia and Canada) was possibly not all captured through the interviews and there may be some challenges or improvement opportunities related to other regions that were not identified (or that do not apply as strongly to other regions). However, the survey with a much larger number of respondents should help mitigate this, especially as there were options for respondents to present additional improvement actions and evidence gaps, and coupled with insights from the literature. Finally, while the survey involved 95 participants from across the five example countries and captured a diversity of perspectives, engagement from participants from France in particular was low (four respondents) limiting the extent to which we could generalize findings in that context.

## Data availability statement

The original contributions presented in this study are included in the article/Supplementary material, further inquiries can be directed to the corresponding author.

## Ethics statement

Ethical approval was not provided for this study on human participants because it was judged to pose minimal risks to participants and not to require ethical approval. The research was conducted in accordance with the Declaration of Helsinki. It was reviewed retrospectively by the RAND Human Subjects Protection Committee and determined to be exempt under 45 CFR 46.104(d)(2)(ii), and, although exempt, the study’s procedures and materials were found by the committee to be consistent with all rules laid out under 45 CFR 46 for the conduct of non-exempt human subjects’ research. This study involved a literature review, interviews with clinical experts and patient representatives, and a survey of clinical experts. All participants gave informed consent and were provided with participant information sheets as part of this process. The patients/participants provided their written informed consent to participate in this study.

## Author contributions

SM and LH were involved in the conception and design of the study. LH, SM, SS, and RR were involved in identifying the sample for data collection, with inputs from PG, TS, JD, NP, and MW. All authors contributed to data collection, analysis, and drafting of the manuscript. All authors approved the contents for publication.
