# Supplementary material for: Improving care for patients with Clostridioides difficile infection: A clinical practice and healthcare systems perspective
Source: Front Med (Lausanne). 2023 Jan 12;9:1033417. doi: 10.3389/fmed.2022.1033417 (PMC9877614; doi:10.3389/fmed.2022.1033417)
Supplement: Supplementary file 1 [file Data_Sheet_1.docx]

Supplementary Material

# Narrative review search termsfc

## Search 1: 5 Case example Countries of Interest

(“Clostridium difficile”[Title/Abstract] OR “C. Diff”[Title/Abstract] OR “C Diff”[Title/Abstract] OR “C. difficile”[Title/Abstract] OR “C-Diff”[Title/Abstract] OR “C-Diff.”[Title/Abstract] OR “C-difficile”[Title/Abstract] OR rCDI[Title/Abstract] OR “Clostridioides difficile”[Title/Abstract] OR “Clostridium infection”[Title/Abstract] OR “Clostridioides infection”[Title/Abstract]) OR (Clostridioides difficile Infection OR Clostridium difficile Infection OR Clostridium difficile Infections OR Clostridium difficile OR Clostridioides difficile[MeSH Terms]))

AND

(diagnos*[Title/Abstract] OR treat*[Title/Abstract] OR manag*[Title/Abstract] OR recur*[Title/Abstract] OR infect*[Title/Abstract] OR healthcare[Title/Abstract] OR “health care”[Title/Abstract] OR care[Title/Abstract] OR “patient experience” [Title/Abstract] OR “experience of patients” [Title/Abstract] OR experience of patient* [Title/Abstract] OR experience of the patient* OR burden[Title/Abstract])

AND

((Australia[Title/Abstract] OR Australian[Title/Abstract] OR Canada[Title/Abstract] OR Canadian[Title/Abstract] OR France[Title/Abstract] OR French[Title/Abstract] OR Italy[Title/Abstract] OR Italian[Title/Abstract] OR "United Kingdom"[Title/Abstract] OR British[Title/Abstract] OR England[Title/Abstract] OR English[Title/Abstract] OR Wales[Title/Abstract] OR Welsh[Title/Abstract] OR Scotland[Title/Abstract] OR Scottish[Title/Abstract] OR "northern Ireland"[Title/Abstract] OR "northern Irish"[Title/Abstract] OR NHS[Title/Abstract] OR “National Health Service”[Title/Abstract]) OR (Australia OR Canada OR France OR Italy OR "United Kingdom" OR England OR Wales OR Scotland OR "northern Ireland"[MeSH Terms]))

Limit to:

- Last 10 years
- English

## Search 2: All High-Income Countries

(“Clostridium difficile”[Title/Abstract] OR “C. Diff”[Title/Abstract] OR “C Diff”[Title/Abstract] OR “C. difficile”[Title/Abstract] OR “C-Diff”[Title/Abstract] OR “C-Diff.”[Title/Abstract] OR “C-difficile”[Title/Abstract] OR rCDI[Title/Abstract] OR “Clostridioides difficile”[Title/Abstract] OR “Clostridium infection”[Title/Abstract] OR “Clostridioides infection”[Title/Abstract]) OR (Clostridioides difficile Infection OR Clostridium difficile Infection OR Clostridium difficile Infections OR Clostridium difficile OR Clostridioides difficile[MeSH Terms]))

AND

(diagnos*[Title/Abstract] OR treat*[Title/Abstract] OR manag*[Title/Abstract] OR recur*[Title/Abstract] OR infect*[Title/Abstract] OR healthcare[Title/Abstract] OR “health care”[Title/Abstract] OR care[Title/Abstract] OR “patient experience” [Title/Abstract] OR “experience of patients” [Title/Abstract] OR experience of patient* [Title/Abstract] OR experience of the patient* OR burden[Title/Abstract])

AND

((Andorra[Title/Abstract] OR Antigua[Title/Abstract] OR Argentina[Title/Abstract] OR Aruba[Title/Abstract] OR Australia[Title/Abstract] OR Austria[Title/Abstract] OR Bahamas[Title/Abstract] OR Bahrain[Title/Abstract] OR Barbados[Title/Abstract] OR Belgium[Title/Abstract] OR Bermuda[Title/Abstract] OR "British Virgin Islands"[Title/Abstract] OR "Brunei Darussalam"[Title/Abstract] OR Canada[Title/Abstract] OR "Cayman Islands"[Title/Abstract] OR "Channel Islands"[Title/Abstract] OR Chile[Title/Abstract] OR Croatia[Title/Abstract] OR Curacao[Title/Abstract] OR Cyprus[Title/Abstract] OR "Czech Republic"[Title/Abstract] OR Denmark[Title/Abstract] OR Estonia[Title/Abstract] OR "Faroe Islands"[Title/Abstract] OR Finland[Title/Abstract] OR France[Title/Abstract] OR "French Polynesia"[Title/Abstract] OR Germany[Title/Abstract] OR Gibraltar[Title/Abstract] OR Greece[Title/Abstract] OR Greenland[Title/Abstract] OR Guam[Title/Abstract] OR Hong Kong[Title/Abstract] OR Hungary[Title/Abstract] OR Iceland[Title/Abstract] OR Ireland[Title/Abstract] OR "Isle of Man"[Title/Abstract] OR Israel[Title/Abstract] OR Italy[Title/Abstract] OR Japan[Title/Abstract] OR Republic of Korea[Title/Abstract] OR Kuwait[Title/Abstract] OR Latvia[Title/Abstract] OR Liechtenstein[Title/Abstract] OR Lithuania[Title/Abstract] OR Luxembourg[Title/Abstract] OR Macao[Title/Abstract] OR Macau[Title/Abstract] OR Malta[Title/Abstract] OR Monaco[Title/Abstract] OR Netherlands[Title/Abstract] OR "New Caledonia"[Title/Abstract] OR "New Zealand"[Title/Abstract] OR "Northern Mariana Islands"[Title/Abstract] OR Norway[Title/Abstract] OR Oman[Title/Abstract] OR Palau[Title/Abstract] OR Panama[Title/Abstract] OR Poland[Title/Abstract] OR Portugal[Title/Abstract] OR "Puerto Rico"[Title/Abstract] OR Qatar[Title/Abstract] OR "San Marino"[Title/Abstract] OR "Saudi Arabia"[Title/Abstract] OR Seychelles[Title/Abstract] OR Singapore[Title/Abstract] OR "Sint Maarten"[Title/Abstract] OR "Slovak Republic"[Title/Abstract] OR Slovakia[Title/Abstract] OR Slovenia[Title/Abstract] OR Spain[Title/Abstract] OR "St. Kitts"[Title/Abstract] OR "St. Martin"[Title/Abstract] OR Sweden[Title/Abstract] OR Switzerland[Title/Abstract] OR Taiwan[Title/Abstract] OR Trinidad[Title/Abstract] OR Turks[Title/Abstract] OR "United Arab Emirates"[Title/Abstract] OR "United Kingdom"[Title/Abstract] OR "United States"[Title/Abstract] OR Uruguay[Title/Abstract] OR "Virgin Islands"[Title/Abstract] OR England*[Title/Abstract] OR Wales[Title/Abstract] OR Scotland[Title/Abstract] OR "northern Ireland"[Title/Abstract] OR NHS[Title/Abstract] OR “national health service”[Title/Abstract]) OR (Andorra OR Antigua OR Argentina OR Aruba OR Australia OR Austria OR Bahamas OR Bahrain OR Barbados OR Belgium OR Bermuda OR "British Virgin Islands" OR "Brunei Darussalam" OR Canada OR "Cayman Islands" OR "Channel Islands" OR Chile OR Croatia OR Curacao OR Cyprus OR "Czech Republic" OR Denmark OR Estonia OR "Faroe Islands" OR Finland OR France OR "French Polynesia" OR Germany OR Gibraltar OR Greece OR Greenland OR Guam OR Hong Kong OR Hungary OR Iceland OR Ireland OR "Isle of Man" OR Israel OR Italy OR Japan OR Republic of Korea OR Kuwait OR Latvia OR Liechtenstein OR Lithuania OR Luxembourg OR Macao OR Macau OR Malta OR Monaco OR Netherlands OR "New Caledonia" OR "New Zealand" OR "Northern Mariana Islands" OR Norway OR Oman OR Palau OR Panama OR Poland OR Portugal OR "Puerto Rico" OR Qatar OR "San Marino" OR "Saudi Arabia" OR Seychelles OR Singapore OR "Sint Maarten" OR "Slovak Republic" OR Slovakia OR Slovenia OR Spain OR "St. Kitts" OR "St. Martin" OR Sweden OR Switzerland OR Taiwan OR Trinidad OR Turks OR "United Arab Emirates" OR "United Kingdom" OR "United States" OR Uruguay OR "Virgin Islands" OR England* OR Wales OR Scotland OR "northern Ireland" [MeSH Terms]))

Limit to:

- Last 5 years
- Review; systematic review; meta-analysis
- English

# Narrative review inclusion/exclusion criteria

| **Criteria** | **Inclusion criteria** | **Exclusion criteria** |
| --- | --- | --- |
| **Publication date** | Priority countries^*^: Published 2011-2021  Other HIC: 2016-2021 | Priority countries: Pre 2011  Other HIC: Pre 2016 |
| **Location** | High income countries, with particular focus on the UK, Italy, France, Australia and Canada. | Non-HIC |
| **Language** | English | Non-English |
| **Study type** | Priority countries: Peer-reviewed journal publications presenting empirical evidence, review papers, grey literature with clear authorship, book chapter, theses, conference proceedings, case reports, regulations, guidance, position statements.  Other HIC: Reviews only. | Documents without clear organisational authorship, theoretical work, letters, editorials, comments or opinion pieces, book reviews. |
| **Diseases** | Harmful *C. difficile* infection (initial and recurrent) | Non-harmful colonisation of *C. difficile.*  Any other disease |
| **Topic** | Healthcare pathway (i.e. diagnosis, treatment, management, referral)- current pathway and improvements  Challenges in the healthcare pathway  Implications of the healthcare pathway in relation to co-morbidities or complications  Evaluation of treatment efficacy (incl. clinical trials of *C. difficile* treatments) studies  Healthcare costs associated with *C. difficile* infection | Cost-effectiveness studies of *C. difficile* treatments  Prevention, transmission, outbreak control in healthcare settings  Biological mechanisms underpinning *C. difficile* infection, treatment or diagnostic methods  Prevalence, mortality, incidence or epidemiology of *C. difficile*  *C. difficile* is not the main focus of the study (unless relevant aspects relating to the healthcare pathway are mentioned)  No abstract (unless title appears highly relevant)  Cause or risk factors of initial infection  Surveillance of *C. difficile* |
| **Study participants** | Humans | Animals and plants |

^*^*Priority countries: UK, France, Italy, Canada and Australia*

### Prioritisation strategy

| **Further considerations to prioritise for REA** |
| --- |
| Including only 2016 onwards |
| Including studies focusing on *C. difficile-* specific populations, e.g. patients with IBD and *C. difficile* (NB: studies focusing on particular care settings, e.g. community, ICU, are still being discussed as to whether they should be included) and age-related populations (e.g. elderly, children). |
| Excluding studies on efficacy of individual *C. difficile* treatments or comparing different treatments (apart from one recent systematic review on treatment efficacy to provide context for the work). |
| Excluding studies on efficacy of individual diagnostic tools |
| Excluding studies conducting clinical trials of interventions, modelling studies or lab studies (i.e. any study which is not about actual care/practice) |
| Excluding *C. difficile* tests or treatment not currently in use |
| Excluding studies not primarily focused on *C. difficile* |
| Prioritising studies focusing on the 5 countries of interest |
| Prioritising studies covering topics that are underrepresented in other included studies and to ensure breadth of topics covered (relevant to the research questions) |

# Preferred reporting items for systematic reviews (PRISMA) diagram for narrative review


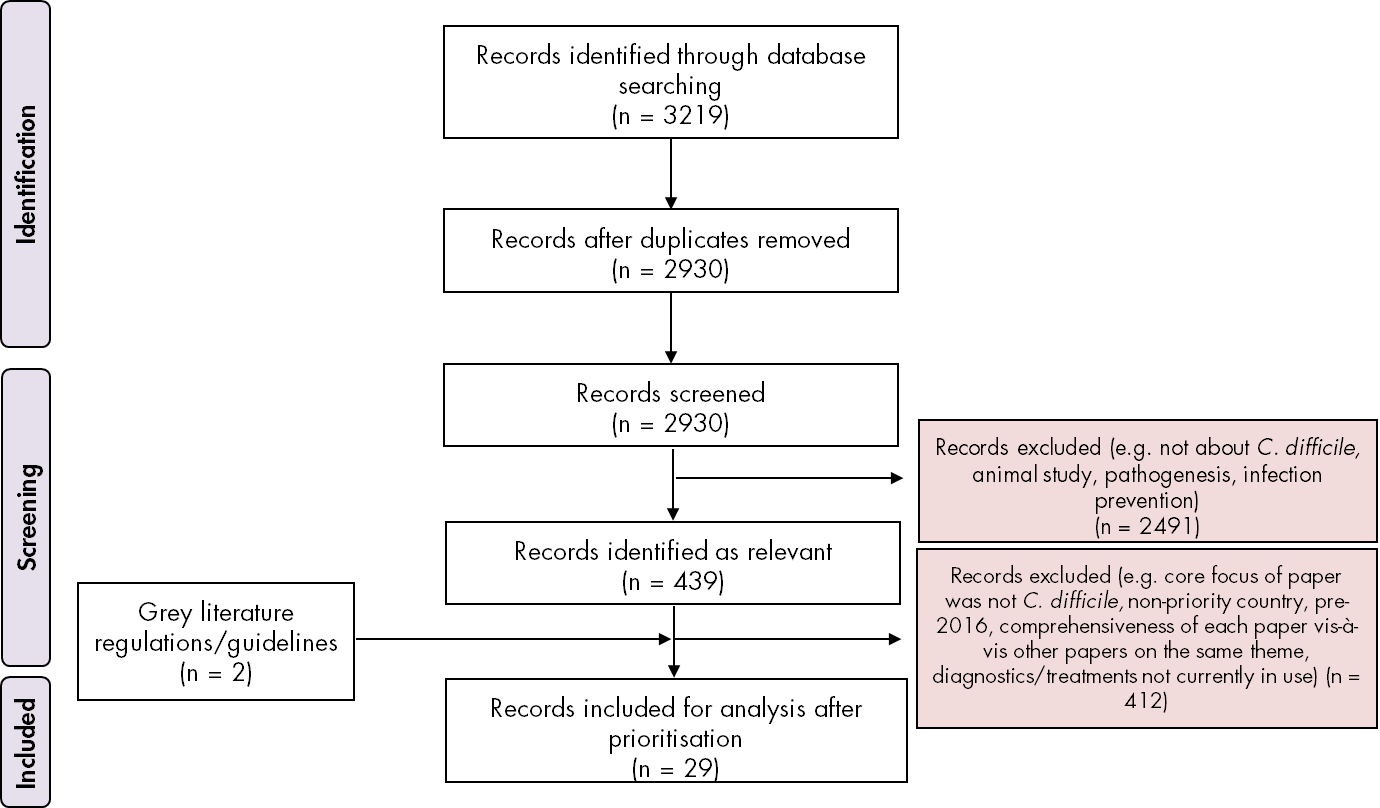


# Consultations with leading clinical experts and patient representatives

Focused discussion was enabled through one-to-one in-depth interviews, conducted by a RAND Europe health services researcher (LH, SS) with leading clinical experts in the case example countries of interest, a total of eight 45-60 minute discussions, conducted between August and December 2021 via Microsoft Teams. Names of experts consulted in one to one interviews s included (in alphabetical order), with informed consent:

- Gail Attara, Canadian Society of Intestinal Research/Gastrointestinal Society (Canada)
- Dr Joel Doré, French National Research Institute for Agriculture, Food and the Environment (France)
- Louise Binder, health advocate (Canada)
- Prof Mark Wilcox, University of Leeds and Leeds Teaching Hospitals NHS Trust (UK)
- Nancy C Caralla, C Diff Foundation (USA)
- Dr Nicola Petrosillo, National Institute for Infectious Diseases Lazzaro Spallanzani (Italy)
- Dr Paul Griffin, University of Queensland (Australia)
- Dr Ted Steiner, The University of British Columbia (Canada)

All interviewees participated with informed consent.

The interviews were semi-structured and followed a topic guide which covered the themes of interest:

- The typical *C. difficile* patient pathway from where the patient makes the first contact with the healthcare system, through to referrals, diagnosis, treatment decisions and ongoing management and monitoring (including CDI recurrences).
- Key challenges along this pathway (for both first episode and recurrent CDI).
- FMT related challenges.
- Challenges in the patient pathway from the viewpoint of patients.
- Impact of COVID-19 on the patient pathway.

Interviews were recorded and detailed interview notes were produced by the research team from RAND Europe, and key themes identified though thematic analysis.

# Survey tool

A note on the survey tool: In this manuscript, we focus on insights related to priorities for improving patient care. The survey also gathered additional information on rating improvement actions (as very, somewhat or not important) which were used to stress test and confirm the ranking findings. We do not go into the detail of the results of these questions in the manuscript to avoid repetitiveness.

Participants were provided links to an information sheet and privacy notice before completing the consent questions.

## Consent

Please complete the following consent questions [yes/no]

- 1. I confirm that I have read the information sheet on participation in the survey
  2. I confirm that I have had the opportunity to consider the information, ask questions and, if applicable, have had these answered satisfactorily.
  3. I understand that my participation is voluntary and that I am free to withdraw at any time without giving any reason, without being penalised in any way and without my legal rights being affected.
  4. I agree to participate in the above survey.
  5. I consent for the survey data to be used by RAND Europe in analysis undertaken as part of the project (all data will be analysed in aggregate, names of individual participants will not be disclosed or passed on to any third party).
  6. I consent to the data being held by RAND Europe for up to one year after the project and thereafter being securely erased.
  7. I consent to being recontacted by a member of the project team to invite me to engage with a second (final) round of the survey and/or if I wish to receive information on aggregated findings.

## Section 1: Respondent profile

2. Which country do you primarily work in?

1. UK
2. France
3. Italy
4. Canada
5. Australia
6. Other

3. Which of the following best describes your clinical area (if more than one, please select the most relevant): *Guidance: we appreciate you may affiliate with more than one area, please select the one that you feel is your primary professional identity*

1. Infectious diseases as primary patient facing focus area
2. Gastroenterology as primary patient facing focus area
3. Elderly/geriatric medicine as a primary patient facing focus area
4. Primary care / general practice / internal medicine / general medicine as primary patient facing focus area
5. Emergency medicine as primary patient facing focus area
6. Microbiology / laboratory science (not necessarily patient-facing work)
7. Pharmacy
8. Other: [please specify*]
9. Which of the following best describes your job role in the clinical area you selected above (if more than one please select the most relevant):
   1. Physician / Medical Doctor
   2. Physician’s Assistant
   3. Nurse
   4. Nurse practitioner
   5. Pharmacist
   6. Microbiology/ laboratory scientist
   7. Microbiology/ laboratory technician
   8. Other clinical role [please specify*]
   9. Other non-clinical role [please specify*]
10. Please provide your full name (first name and surname)
11. Please provide a contact email so that we could share the feedback/learning from the survey with you and give you the opportunity to participate in the second and final prioritisation survey round

## Section 2: Improvement actions and gaps in evidence

This section covers potential improvement actions that are directly related to clinical practices in the diagnosis and treatment of patients, such as those related to the nature of available diagnostics, treatments and decision-making tools. We then consider improvement opportunities related to the wider nature of healthcare systems, such as improvement opportunities related to access to care and the organisation and delivery of healthcare, regulation and guidelines, and education and awareness raising needs.

### Part I – Improvement actions: Diagnosis and treatment

1. Based on your professional expertise, please rate the importance of each of the following **improvement actions** as they relate to the **diagnosis and treatment of *C.* *difficile* infection.** *Guidance: Please rate each row (i.e. each improvement need)*

| **Improvement actions** | **I do not know / have an opinion** | **Not important** | **Somewhat important** | **Very important** |
| --- | --- | --- | --- | --- |
| 1. **Diagnosis**: Improve the **accuracy** of diagnostic methods through innovation |  |  |  |  |
| 1. **Diagnosis**: Improve the **speed** of diagnostic methods through innovation |  |  |  |  |
| 1. **Diagnosis:** Develop protocols and policies for lab staff to use to inform decisions about which tests to use and in what order for patients who need diagnostic testing for *C. difficile* |  |  |  |  |
| 1. **Treatment:** Develop innovative, more effective treatments for **treating initial *C. difficile* infection** compared with current treatment options |  |  |  |  |
| 1. **Treatment**: Develop innovative, more effective treatments for **treating recurrent *C. difficile* infection** compared with current treatment options |  |  |  |  |
| 1. **Prevention of recurrence:** Develop innovative products for **preventing recurrence** of C. difficile infection |  |  |  |  |
| 1. **Disease classification**: Obtain consensus on definitions and classifications relating to *C.* *difficile* infection, such as classifications of severity of disease, definitions of recurrence in support of improving disease management and patient outcomes and/or more efficiently using healthcare resources |  |  |  |  |
| 1. **Disease classification**: Establish better methods to distinguish recurrence from a new *C.* *difficile* infection |  |  |  |  |
| 1. **Prediction of recurrence**: Develop algorithms to more accurately predict the risk of *C.* *difficile* infection recurrences, based on patient and clinical profile, in support of earlier intervention, improving patient outcomes and/or more efficiently using healthcare resources |  |  |  |  |
| 1. **Reaching decisions about patient cure:** Establish better methods to define and test for cure of *C.* *difficile* infection, in support of improving patient outcomes and/or more efficiently using healthcare resources |  |  |  |  |

1. Please select up to **3 improvement actions** related to **diagnosis and treatment of CDI** that you think are the most important within this list. *Guidance: Please only select up to 3*

| **Improvement actions** | **Tick up to 3** |
| --- | --- |
| 1. **Diagnosis**: Improve the **accuracy** of diagnostic methods through innovation |  |
| 1. **Diagnosis**: Improve the **speed** of diagnostic methods through innovation |  |
| 1. **Diagnosis:** Develop protocols and policies for lab staff to use to inform decisions about which tests to use and in what order for patients who need diagnostic testing for *C. difficile* |  |
| 1. **Treatment:** Develop innovative, more effective treatments for **treating initial *C. difficile* infection** compared with current treatment options |  |
| 1. **Treatment**: Develop innovative, more effective treatments for **treating recurrent *C. difficile* infection** compared with current treatment options |  |
| 1. **Prevention of recurrence:** Develop innovative products for **preventing recurrence** of C. difficile infection |  |
| 1. **Disease classification**: Obtain consensus on definitions and classifications relating to *C.* *difficile* infection, such as classifications of severity of disease, definitions of recurrence in support of improving disease management and patient outcomes and/or more efficiently using healthcare resources |  |
| 1. **Disease classification**: Establish better methods to distinguish recurrence from a new *C.* *difficile* infection |  |
| 1. **Prediction of recurrence**: Develop algorithms to more accurately predict the risk of *C.* *difficile* infection recurrences, based on patient and clinical profile, in support of earlier intervention, improving patient outcomes and/or more efficiently using healthcare resources |  |
| 1. **Reaching decisions about patient cure:** Establish better methods to define and test for cure of *C.* *difficile* infection, in support of improving patient outcomes and/or more efficiently using healthcare resources |  |
| 1. None of the above |  |

### Part II – Improvement actions: Access, organisation of service delivery and quality of care

1. Based on your professional expertise, please rate the importance of each of the following **improvement actions** as they relate to the **organisation of service delivery and quality of care for patients with *C.* *difficile* infection.** *Guidance: Please rate each row (i.e. each improvement need)*

| **Improvement actions** | **I do not know / have an opinion** | **Not important** | **Somewhat important** | **Very important** |
| --- | --- | --- | --- | --- |
| 1. **Timeliness of care**: Improve timeliness of diagnosis (e.g. timely referrals for testing, timely turnaround of test conduct and results) |  |  |  |  |
| 1. **Access to Faecal Microbiota Transplantation:** Address variation in access to Faecal Microbiota Transplantation at local, regional and national levels (e.g. though interventions which can improve availability of the procedure and/or optimise referral behaviours across clinical specialties (e.g. gastroenterologists, infectious disease specialists, primary care)) |  |  |  |  |
| 1. **Access to antibiotics:** Improve access to effective antibiotics for treating *C*. *difficile* infection |  |  |  |  |
| 1. **Access:** Improve patient access to monoclonal antibody therapy for preventing and/or treating *C.* *difficile* infection recurrences) |  |  |  |  |
| 1. **Access**: Improve mental health support services for patients with *C.* *difficile* infection to mitigate against psychological distress that is associated with the course of illness |  |  |  |  |
| 1. **Organisation of care**: Facilitate more multi-disciplinary care delivery in the management of patients with *C.* *difficile* infection (e.g. across infectious disease specialists, gastroenterologists, microbiologists and other healthcare professionals) to support joined-up decision making and involvement of necessary expertise |  |  |  |  |

1. Please select up to **2** **improvement actions** related to **the organisation of service delivery and quality of care for *C.* *difficile* infection** that you think are the most important within this list. *Guidance: Please only select up to 2*

| **Improvement actions** | **Tick up to 2** |
| --- | --- |
| 1. **Timeliness of care**: Improve timeliness of diagnosis (e.g. timely referrals for testing, timely turnaround of test conduct and results) |  |
| 1. **Access to Faecal Microbiota Transplantation:** Address variation in access to Faecal Microbiota Transplantation at local, regional and national levels (e.g. though interventions which can improve availability of the procedure and/or optimise referral behaviours across clinical specialties (e.g. gastroenterologists, infectious disease specialists, primary care)) |  |
| 1. **Access to antibiotics:** Improve access to effective antibiotics for treating *C*. *difficile* infection |  |
| 1. **Access:** Improve patient access to monoclonal antibody therapy for preventing and/or treating *C.* *difficile* infection recurrences) |  |
| 1. **Access**: Improve mental health support services for patients with *C.* *difficile* infection to mitigate against psychological distress that is associated with the course of illness |  |
| 1. **Organisation of care**: Facilitate more multi-disciplinary care delivery in the management of patients with *C.* *difficile* infection (e.g. across infectious disease specialists, gastroenterologists, microbiologists and other healthcare professionals) to support joined-up decision making and involvement of necessary expertise |  |
| 1. None of the above |  |

### Part III – Improvement actions: Guidelines and regulation

1. Based on your professional expertise, please rate the importance of each of the following **improvement actions** as they relate to **guidelines and regulation for *C. difficile* infection treatments.** *Guidance: Please rate each row (i.e. each improvement need)*

| **Improvement actions** | **I do not know / have an opinion** | **Not important** | **Somewhat important** | **Very important** |
| --- | --- | --- | --- | --- |
| 1. **Guidelines:** Update diagnosis and treatment guidelines more regularly in light of new research so that clinicians have the most relevant, evidence-based recommendations to support decision-making. |  |  |  |  |
| 1. **Guidelines:** Standardise **diagnosis guidelines** within countries to reduce unwarranted variation in care across regions/localities (what test to use, what order) |  |  |  |  |
| 1. **Guidelines:** Standardise **treatment guidelines** within countries to reduce unwarranted variation in care across regions/localities |  |  |  |  |
| 1. **Guidelines:** Reduce unwarranted variation through standardisation of Faecal Microbiota Transplantation practice, supported by implementing standardised protocols to ensure safety, efficacy and adherence to quality standards |  |  |  |  |
| 1. **Guidelines:** Incorporate information on cost-effectiveness of specific treatments into guidelines so that the local feasibility of guideline implementation can be assessed |  |  |  |  |
| 1. **Guidelines:** Improve clarity of guidelines (in length and tone) so that communications are clear and more digestible to busy clinicians |  |  |  |  |
| 1. **Wider regulation:** Remove regulatory barriers to access innovative diagnostic and treatment options for *C. difficile* (e.g. access to drugs outside of clinical trials, classification of treatments) |  |  |  |  |
| 1. **COVID-19:** Establish clear policies and practices for managing patients with *C. difficile* during the COVID-19 pandemic |  |  |  |  |
| 1. **Local policies:** Ensure that local hospital committees have clear policies regarding what therapies/services are available and can be accessed by patients (as related to *C. difficile)* and under which conditions/criteria |  |  |  |  |

1. Please select up to **3** **improvement actions** related to **guidelines and regulation for CDI treatments** that you think are the most important. *Guidance: Please only select up to 3*

| **Improvement actions** | **Tick up to 3** |
| --- | --- |
| 1. **Guidelines:** Update diagnosis and treatment guidelines more regularly in light of new research so that clinicians have the most relevant, evidence-based recommendations to support decision-making. |  |
| 1. **Guidelines:** Standardise **diagnosis guidelines** within countries to reduce unwarranted variation in care across regions/localities (what test to use, what order) |  |
| 1. **Guidelines:** Standardise **treatment guidelines** within countries to reduce unwarranted variation in care across regions/localities |  |
| 1. **Guidelines:** Reduce unwarranted variation through standardisation of Faecal Microbiota Transplantation practice, supported by implementing standardised protocols to ensure safety, efficacy and adherence to quality standards |  |
| 1. **Guidelines:** Incorporate information on cost-effectiveness of specific treatments into guidelines so that the local feasibility of guideline implementation can be assessed |  |
| 1. **Guidelines:** Improve clarity of guidelines (in length and tone) so that communications are clear and more digestible to busy clinicians |  |
| 1. **Wider regulation:** Remove regulatory barriers to access innovative diagnostic and treatment options for *C. difficile* (e.g. access to drugs outside of clinical trials, classification of treatments) |  |
| 1. **COVID-19:** Establish clear policies and practices for managing patients with *C. difficile* during the COVID-19 pandemic |  |
| 1. **Local policies:** Ensure that local hospital committees have clear policies regarding what therapies/services are available and can be accessed by patients (as related to *C. difficile)* and under which conditions/criteria |  |
| 1. None of the above |  |

### Part IV – Improvement actions: Education and awareness-raising for patients

1. Based on your professional expertise, please rate the importance of each of the following **improvement actions** as they relate to ***C. difficile* infection** **education and awareness-raising of patients.** *Guidance: Please rate each row (i.e. each improvement need)*

| **Improvement actions** | **I do not know / have an opinion** | **Not important** | **Somewhat important** | **Very important** |
| --- | --- | --- | --- | --- |
| 1. **Patient education and awareness:** Raise awareness about *C.* *difficile* infection amongst the public through government funded or other campaigns (e.g. raise awareness on issues related to risks of infection, symptoms, importance of patients not feeling embarrassed to seek support from healthcare professionals to enable timelier diagnosis) |  |  |  |  |
| 1. **Patient education and awareness:** Educate patients with *C.* *difficile* infection about the management of the illness and the potential future impact on their lives (e.g. lifestyle changes, being more aware of toileting habits/bowel movements, psychological and physiological aspects of condition, risks of recurrence) - for example, through various national or local educational campaigns and through healthcare professionals providing educational material and information to patients |  |  |  |  |
| 1. **Patient education and awareness:** Educate patients with *C.* *difficile* infection on the appropriate use of antibiotics (i.e. to ensure patients don’t stop taking antibiotics for *C. difficile* infection prior to completing a course) to mitigate against the development of treatment-resistant strains of *C.* *difficile* or recurrence |  |  |  |  |
| 1. **Patient education and awareness:** Educate patients on the current state of evidence related to the use of probiotics in treatment/prevention of *C.* *difficile* infection |  |  |  |  |
| 1. **Patient education and awareness:** Create *C.* *difficile* infection-specific patient organisations as a support and information sharing network |  |  |  |  |
| 1. **Patient education and awareness:** Better disseminate existing educational resources to patients |  |  |  |  |
| 1. **Patient choice and education related to access to Faecal Microbiota Transplantation:** Improve patient choice in relation to Faecal Microbiota Transplantation as a treatment option through education, awareness raising and information provision |  |  |  |  |

1. Please select up to **3 improvement actions** related to **education and awareness-raising** that you think are the most important. *Guidance: Please only select up to 3*

| **Improvement actions** | **Tick up to 3** |
| --- | --- |
| 1. **Patient education and awareness:** Raise awareness about *C.* *difficile* infection amongst the public through government funded or other campaigns (e.g. raise awareness on issues related to risks of infection, symptoms, importance of patients not feeling embarrassed to seek support from healthcare professionals to enable timelier diagnosis) |  |
| 1. **Patient education and awareness:** Educate patients with *C.* *difficile* infection about the management of the illness and the potential future impact on their lives (e.g. lifestyle changes, being more aware of toileting habits/bowel movements, psychological and physiological aspects of condition, risks of recurrence) - for example, through various national or local educational campaigns and through healthcare professionals providing educational material and information to patients |  |
| 1. **Patient education and awareness:** Educate patients with *C.* *difficile* infection on the appropriate use of antibiotics (i.e. to ensure patients don’t stop taking antibiotics for *C. difficile* infection prior to completing a course) to mitigate against the development of treatment-resistant strains of *C.* *difficile* or recurrence |  |
| 1. **Patient education and awareness:** Educate patients on the current state of evidence related to the use of probiotics in treatment/prevention of *C.* *difficile* infection |  |
| 1. **Patient education and awareness:** Create *C.* *difficile* infection-specific patient organisations as a support and information sharing network |  |
| 1. **Patient education and awareness:** Better disseminate existing educational resources to patients |  |
| 1. **Patient choice and education related to access to Faecal Microbiota Transplantation:** Improve patient choice in relation to Faecal Microbiota Transplantation as a treatment option through education, awareness raising and information provision |  |
| 1. None of the above |  |

### Part V – Improvement actions: Education and awareness-raising for clinicians

1. Based on your professional expertise, please rate the importance of each of the following **improvement actions** as they relate to ***C. difficile infection*** **education and awareness-raising of clinicians.** *Guidance: Please rate each row (i.e. each improvement need)*

| **Improvement actions** | **I do not know / have an opinion** | **Not important** | **Somewhat important** | **Very important** |
| --- | --- | --- | --- | --- |
| 1. **Healthcare professional education and awareness:** Educate and support healthcare professionals **in primary care** on identifying *C.* *difficile* infection symptoms, when and how to test and diagnose patients with *C.* *difficile* infection (or refer for testing and treatment to a specialist) and how to manage patients who are being treated |  |  |  |  |
| 1. **Healthcare professional education and awareness:** Educate and support healthcare professionals in secondary care (i.e. hospitals) who are not experts regularly dealing with patients with *C.* *difficile* infections (e.g. A&E staff, surgeons treating patients, ICU staff, oncology staff) on identifying *C.* *difficile* infection symptoms, when and how to refer for testing for *C.* *difficile* infection and how to manage patients under their care who may have *C.* *difficile* |  |  |  |  |
| 1. **Healthcare professional education and awareness:** Educate and support healthcare professionals in primary and secondary care on the optimal approach to treat recurrent or refractory (persistent) CDI to reduce variability and to reduce the use of non-evidence based practice |  |  |  |  |
| 1. **Healthcare professional education and awareness:** Education for pharmacists around good antimicrobial stewardship for *C.* *difficile* infection to ensure only antibiotics known to treat *C.* *difficile* infection effectively are prescribed |  |  |  |  |
| 1. **Healthcare professional education and awareness:** Set up networks of expert clinicians as a resource for other healthcare professionals to seek advice on managing *C.* *difficile* infection patients and for sharing experiences and learning |  |  |  |  |
| 1. **Healthcare professional education and awareness:** Provide information and educational support for healthcare professionals on how to effectively and confidently engage with patients in terms of making patients feel comfortable to discuss *C.* *difficile* symptoms and to help them combat the stigma/embarrassment factor |  |  |  |  |
| 1. **Healthcare provider education and awareness:** Improve healthcare provider knowledge and training around Faecal Microbiota Transplantation to build greater future workforce capacity to deliver a safe and effective service (e.g. by integrating education about Faecal Microbiota Transplantation into medical education curricula and professional development) |  |  |  |  |

1. Please select up to **3 improvement actions** related to **education and awareness-raising** that you think are the most important. *Guidance: Please only select up to 3*

| **Improvement actions** | **Tick up to 2** |
| --- | --- |
| 1. **Healthcare professional education and awareness:** Educate and support healthcare professionals **in primary care** on identifying *C.* *difficile* infection symptoms, when and how to test and diagnose patients with *C.* *difficile* infection (or refer for testing and treatment to a specialist) and how to manage patients who are being treated |  |
| 1. **Healthcare professional education and awareness:** Educate and support healthcare professionals in secondary care (i.e. hospitals) who are not experts regularly dealing with patients with *C.* *difficile* infections (e.g. A&E staff, surgeons treating patients, ICU staff, oncology staff) on identifying *C.* *difficile* infection symptoms, when and how to refer for testing for *C.* *difficile* infection and how to manage patients under their care who may have *C.* *difficile* |  |
| 1. **Healthcare professional education and awareness:** Educate and support healthcare professionals in primary and secondary care on the optimal approach to treat recurrent or refractory (persistent) CDI to reduce variability and to reduce the use of non-evidence based practice |  |
| 1. **Healthcare professional education and awareness:** Education for pharmacists around good antimicrobial stewardship for *C.* *difficile* infection to ensure only antibiotics known to treat *C.* *difficile* infection effectively are prescribed |  |
| 1. **Healthcare professional education and awareness:** Set up networks of expert clinicians as a resource for other healthcare professionals to seek advice on managing *C.* *difficile* infection patients and for sharing experiences and learning |  |
| 1. **Healthcare professional education and awareness:** Provide information and educational support for healthcare professionals on how to effectively and confidently engage with patients in terms of making patients feel comfortable to discuss *C.* *difficile* symptoms and to help them combat the stigma/embarrassment factor |  |
| 1. **Healthcare provider education and awareness:** Improve healthcare provider knowledge and training around Faecal Microbiota Transplantation to build greater future workforce capacity to deliver a safe and effective service (e.g. by integrating education about Faecal Microbiota Transplantation into medical education curricula and professional development) |  |
| 1. None of the above |  |

## Section 3: Gaps in evidence

We are now moving on to ask which areas of *C. difficile*  research you think are most in need of further evidence. You will be given the opportunity to share any additional evidence gaps that exist in the following section.

1. Please select up to 3 evidence gaps that you think are the most important to address. *Guidance: Please only select up to 3*

| **Gaps in evidence** | **Tick up to 3** |
| --- | --- |
| 1. **Treatment:** Better evidence on optimal treatment regimens in managing patients with specific profiles (e.g., severe CDI, older age, those with chronic comorbidities) and for preventing and treating recurrences in these patients |  |
| 1. **Treatment:** Better evidence on the **long-term** effectiveness and safety of existing treatment options |  |
| 1. **Treatment:** Better evidence on the efficacy and safety of emerging preventatives or treatments (currently available but novel or still in development), such as monoclonal antibody therapy and/or probiotics. |  |
| 1. **Treatment:** Better evidence on the threat of antimicrobial resistance in the treatment of C. *difficile* infection |  |
| 1. **Treatment:** Better evidence on whether monoclonal antibody therapy can be used as a treatment rather than just preventative method |  |
| 1. **Economic considerations/cost-effectiveness:** Better evidence on the cost-effectiveness of treatments in diverse geographical settings (i.e., what is cost-effective in one setting, may not be cost-effective in others given different healthcare system financing approaches and costs of service provision) |  |
| 1. **Risk factors and patient profile:** Better evidence on risk factors associated with recurrence |  |
| 1. **Patient experience:** Improved evidence and understanding of patient perspectives on C. *difficile* infection and their care needs, including impact on quality of life |  |
| 1. **Regulation:** Improved evidence on the extent of variation in healthcare professional adherence to diagnosis and treatment guidelines and where action could be taken to reduce unwarranted variation |  |
| 1. None of the above |  |

## Section 4: Other improvement actions or evidence gaps

1. Are there any **improvement actions** related to the care of patients with *C.* *difficile* infection that have not been mentioned and that you think are very important? If so, please explain
2. Are there any other **evidence gaps** related to the care of patients with *C.* *difficile* infection that were not mentioned in the list above that you think are very important? If so, please explain.

## Section 5: The *C. difficile* patient pathway

1. In terms of patients with **community acquired** *C. difficile* infection: If a patient in your country has a community acquired *C. difficile* infection, who is most likely to be the first point of contact for the patient in the healthcare system in relation to their symptoms?
   1. A primary care professional (e.g. primary care physician, nurse)
   2. An infectious disease expert in an outpatient hospital setting
   3. A gastroenterologist expert in an outpatient hospital setting
   4. A healthcare professional in emergency care setting
   5. There is not one particularly common/predominant point of first contact for patients with the healthcare system
   6. Other, please specify
   7. I do not know
2. In terms of patients with **hospital acquired** *C. difficile* infection: If a patient in your country who is in hospital for other reasons (i.e. reasons that are not originally *C. difficile* related) develops symptoms of a hospital acquired *C. difficile* infection, who will first see them specifically related to the *C. difficile* care decisions:
   1. An infectious disease expert
   2. A gastroenterologist expert
   3. The person under whose care they are for the other condition will be the one to order *C. difficile* tests
   4. There is no one particularly common/predominant type of expert who they will see for *C. difficile* related care
   5. Other, please specify
   6. I do not know
3. **If this patient’s first point of contact with the healthcare system is within a primary care or outpatient setting: If further care related to *C. difficile* is needed, who would the initial healthcare professional most likely refer the patient on to next?** Would they most often be referred to:
   1. An infectious disease expert in an outpatient setting
   2. A gastroenterologist in an outpatient setting
   3. The emergency department in a hospital
   4. Infection prevention and control nurse/team
   5. *C. difficile* infection multidisciplinary team
   6. Other primary care professional
   7. They are likely to be receive referrals to multiple healthcare professionals at the same time (if so, please specify types of healthcare professionals they would be referred to)
   8. There is no one predominant person/care setting they would be referred to as it depends on severity of symptoms, parts of the country, preferences and experience of the person doing the referral etc.
   9. They most likely not be referred to anyone further- the healthcare professional they first see is likely to continue with decisions related to their *C. difficile* related care needs
   10. Other, please specify
   11. I do not know
4. **If this patient’s first point of contact with the healthcare system is within an inpatient/hospital admission setting: If further care related to *C. difficile* is needed, who would the initial healthcare professional most likely refer the patient on to next?** Would they most often be referred to:
   1. An infectious disease expert in an inpatient setting
   2. A gastroenterologist in an inpatient setting
   3. The emergency department in a hospital
   4. Infection prevention and control nurse/team
   5. *C. difficile* infection multidisciplinary team
   6. A primary care professional
   7. They are likely to be receive referrals to multiple healthcare professionals at the same time (if so, please specify types of healthcare professionals they would be referred to)
   8. There is no one predominant person/care setting they would be referred to as it depends on severity of symptoms, parts of the country, preferences and experience of the person doing the referral etc.
   9. They most likely not be referred to anyone further- the healthcare professional they first see is likely to continue with decisions related to their *C. difficile* related care needs
   10. Other, please specify
   11. I do not know

# Demographics of survey respondents

Table 1: Survey respondent demographics

| Demographic | Number (%) |
| --- | --- |
| **Country** | |
| Italy | 38 (40.0%) |
| UK | 25 (26.3%) |
| Australia | 16 (16.8%) |
| Canada | 12 (12.6%) |
| France | 4 (4.2%) |
| **TOTAL** | **95 (100%)** |
| **Clinical area** | |
| Infectious diseases as primary patient facing focus area | 58 (61.1%) |
| Other | 10 (10.5%) |
| Microbiology / laboratory science (not necessarily patient-facing work) | 10 (10.5%) |
| Gastroenterology as primary patient facing focus area | 8 (8.4%) |
| Primary care/general practice/internal medicine/general medicine as primary patient facing focus area | 6 (6.3%) |
| Emergency medicine as primary patient facing focus area | 1 (1.1%) |
| Pharmacy | 1 (1.1%) |
| Elderly/geriatric medicine as a primary patient facing focus area | 1 (1.1%) |
| **TOTAL** | **95 (100%)** |
| **Job role** | |
| Physician/Medical Doctor | 78 (82.1%) |
| Nurse | 9 (9.5%) |
| Nurse practitioner | 2 (2.1%) |
| Pharmacist | 2 (2.1%) |
| Microbiology/laboratory scientist | 2 (2.1%) |
| Other | 2 (2.1%) |
| **TOTAL** | **95 (100%)** |

# Further information on referral pathways from survey data

In most of the case example countries, survey data suggests that the first point of contact with the healthcare system for the majority of patients with community acquired CDI infections is a primary care professional (92% of survey respondents in the UK, 88% in Australia, 67% in Canada and 61% in Italy), although this was not the case for France (0%, although only 4 respondents were from France). This was also supported by evidence from expert interviewees (INT-7). Some survey respondents from Canada (8%) identified emergency care settings as a primary point of contact for community acquired CDI, most likely in cases of severe symptoms. In Italy, patients can also present to emergency care settings or to infectious disease (ID) experts in outpatient hospital settings, for example if diarrhea is severe (18% and 11% of survey respondents from Italy, respectively). The presentation at emergency settings in Italy was also raised by the expert interviewee who discussed how this may be to lack of confidence in primary care physicians from the public and lack of primary care provider awareness of CDI (INT3). Although rarely, in the UK, a patient with community acquired infection may also first present to a community-based infection prevention and control team (selected as primary point of contact by 4% of survey respondents). Some respondents (50% in France, 17% in Canada, 8% in Italy, 6% in Australia, 4% in UK) felt that there was not one predominant point of contact with the healthcare system, reflecting diverse practices regionally or different patient symptoms-related factors. In the UK, the primary point of contact was also noted to vary depending on patient specific factors by 4% of survey respondents and 4% noted that community can also be a primary point of contact with the healthcare system. The first point of contact in primary care tends to order diagnostic tests in the UK. In France, 25% of respondents (2 respondents) reported that gastroenterologist expert in an outpatient hospital setting was the primary point of contact.

For patients with hospital acquired CDI in Australia, Canada, the UK and France, the first point of care for patients will be the person under whose care they are more generally (81%, 75%, 80% and 50% of respondents, respectively). In Italy, the situation differs with the primary physician under whose care an inpatient is being selected as the most common route by only 40% of survey respondents. More common in Italy was referral to an ID expert (47%), while this option being rarer in France, Australia, the UK and Canada (25%, 19%, 16% and 8% respectively). In Canada and Italy, patients with hospital acquired infection can also be seen by gastroenterologists (GIs) as a first point of contact (8% each). In Italy, a small number of respondents noted there is no one predominant practice in terms of who will see the patient first (3%). In the UK, a small minority of survey respondents noted that individuals with hospital acquired infection can also initially see a nursing team (4%).

Who the patient will be referred to from the first point of contact in a community settings seems to vary both within and between countries. For example, in Australia 50% of survey respondents noted that referral will tend to be to ID experts in outpatient settings but can also be to a GI (19%) and sometimes there may not be any onward referral (6%). In Canada, the most common onward referral route was said to be to an ID expert (75%), with occasional referral to GI (8%). In Italy, just under half of survey respondents noted referrals to go primarily to ID experts in an outpatient setting (47%), just over a fifth reported the most common referral route was to an emergency care setting (21%), 6% noted there is likely to not be an onward referral, 3% noted referrals to multidisciplinary teams, and – unlike in Australia, Canada and the UK - no respondents selected GI as the onward point of referral. In the UK, the survey data points to particularly variable practice; 16% of respondents noted most common referral to be to a GI and none selected onward referral to an ID specialist as common unlike in Australia, Canada and Italy, although 16% noted referral to microbiologists in hospital settings and these are likely to work closely with ID experts. In addition, 12% of UK respondents noted referral to infection prevention and control nurses/teams, 8% to emergency departments, 4% each a severe disease surgical referral and 4% a referral to another primary care professional. A further 8% noted that the patient is most likely not to be referred to anyone further and that the healthcare professional they first see is likely to continue with decisions related to their CDI related care needs. In addition, four UK respondents stated that patients would be referred to a microbiologist. In France, 25% of respondents noted that a patient would be referred to a GI in an outpatient setting and 25% to a CDI multidisciplinary team. 25% of survey respondents in Australia, 24% in the UK, 18% in Italy and 8% in Canada flagged that there is no one predominant referral route and that this will depend on factors such as the severity of patient symptoms, parts of the country and preferences and personal experiences of the referring healthcare professional.

In terms of onward referral from inpatient/hospital admission settings, in Australia, Canada and Italy this is most often to an ID specialist in the inpatient setting (69%, 58% and 74%, respectively) and less commonly to other experts such as GI (13%, 17%, 8%, respectively) or patients receiving referrals to multiple healthcare professionals at the same time (6%, 8%, 5% respectively). In Australia and Canada, patients can also receive onward referrals to infection prevention and control nurses/teams (6% and 8%, respectively). In Italy, Australia and the UK, there can also be no one predominant expert onwards referral, although in a minority of cases (8%, 6% and 4% respectively). In France, 50% of respondents noted that onward referral would be to GIs, and 25% to ID expert in an inpatient setting. The UK survey data paints a picture of large variety in practices with infection prevention and control nurses/teams being selected by 28% of respondents as the most likely point of onward referral, GI in inpatient setting by 24% of respondents and ID experts by 20%. Other options were also identified as examples (4% of survey respondents each) such as onward referral to combinations of experts in microbiology, infection control and possibly ID or microbiology and ID or microbiology potentially in combination with GIs or surgeons depending on clinical scenarios.

# Summary of challenges

The challenges associated with the care of patients with CDI span clinical practice and wider healthcare system level issues. Table 2 complements summarizes the key challenges applying to the case-example country contexts, based on expert consultation.

Table 2: Challenges in the patient pathway (grey highlighted rows represent most common challenges across the example countries)

|  | **UK** | **Italy** | **France** | **Canada** | **Australia** |
| --- | --- | --- | --- | --- | --- |
| Diagnosis of CDI |  |  |  |  |  |
| Long wait times for diagnosis, e.g. diagnosis taking longer for patients in the community compared to inpatients | ✓ |  | ✓ | ✓ | ✓ |
| Across regions within a country, different testing algorithms are used due to differences in guidelines and so there is a lack of standardized practice and guidelines | ✓ | ✓ |  | ✓ | ✓ |
| There is no single test that is recommended to be used for diagnosis alone and different tests come with advantages and disadvantages related to accuracy, turnaround time and distinguishing colonization from toxigenic infection; the frequent use of multiple tests to arrive at a diagnosis has both time and cost implications | ✓ | ✓ | ✓ | ✓ | ✓ |
| CDI can be overdiagnosed in hospitals due to, e.g. a requirement to test all inpatient cases of diarrhea for CDI, false positive test results or over testing due to performance management requirements | ✓ | ✓ |  | ✓ |  |
| Misdiagnosis of CDI can occur, e.g. in testing after treatment which can arise in a false positive due to *C. difficile* material remaining in stool or difficulties distinguishing between *C. difficile* colonization and toxigenic infection in some patient groups | ✓ | ✓ |  | ✓ | ✓ |
| Treatment of first episode CDI |  |  |  |  |  |
| Treatments for CDI can cause undesirable side effects, such as antibiotics misbalancing the gut microbiome further. | ✓ | ✓ | ✓ | ✓ | ✓ |
| Patients with additional complexities, such as the elderly and patients with co-morbidities, may face difficulties in treating their CDI due to frailty, multiple health issues that need addressing or a lack of response to treatment | ✓ |  |  | ✓ |  |
| Patient monitoring and follow-up |  |  |  |  |  |
| Monitoring CDI patients in hospital can be difficult as bowel movements are not always easy to record due to lack of available staff or threshold of 3+ loose bowel movements for a patient to be tested for CDI | ✓ |  |  |  |  |
| It is difficult to know when a patient is ‘cured’ |  |  |  |  | ✓ |
| Managing CDI recurrences |  |  |  |  |  |
| CDI recurrences can be challenging to diagnose, e.g. due to lack of monitoring for recurrence symptoms | ✓ | ✓ |  | ✓ | ✓ |
| While FMT is generally considered safe, there are some risks of adverse events and there are some concerns about the lack of research into long-term safety | ✓ |  |  |  |  |
| Workforce capacity, facilities and resource challenges can impact on access to FMT |  |  |  | ✓ |  |
| Access and organization of service delivery and quality of care |  |  |  |  |  |
| Donor recruitment is a challenge due to a lack of reimbursement, donor fatigue and low number of eligible donors |  |  | ✓ | ✓ | ✓ |
| Access to FMT can be a challenge, e.g. for patients in rural areas | ✓ |  | ✓ | ✓ | ✓ |
| There is a lack of standardization of FMT procedures and a need for further evidence on optimal stool preparation procedures and modes of FMT delivery (e.g. colonoscopy, enema, capsules) which can also be barriers to optimal patient care and experiences | ✓ | ✓ |  | ✓ |  |
| The organization of healthcare services patients with CDI, such as links between primary and secondary care, the set-up of outpatient care and availability of specialist CDI clinics, can influence the type of care CDI patients receive | ✓ | ✓ |  |  | ✓ |
| There can also be variety in the degree of and multidisciplinary working which can influence the care of some patients with CDI | ✓ |  | ✓ |  | ✓ |
| Guidelines and regulations |  |  |  |  |  |
| Challenges with guideline clarity | ✓ |  | ✓ |  |  |
| While guidelines may be in place to support the treatment and management of patients with CDI in many countries, evidence suggests that these are not updated on a regular basis which is a challenge to optimizing care quality. | ✓ | ✓ |  | ✓ | ✓ |
| Guidelines are also often modified or applied inconsistently in clinical practice, compromising standardized practice. (Whether this is in some cases warranted merits further research) |  | ✓ |  |  |  |
| Economic considerations |  |  |  |  |  |
| The cost of some antibiotics may be difficult for healthcare systems to absorb and this may also be a challenge in relation to emerging treatments as well as in relation to treating recurrence | ✓ | ✓ | ✓ | ✓ | ✓ |
| Cost can be a barrier not only to optimizing treatment, but also to optimal diagnostic test use and may contributes to some of the variation seen in CDI guidelines across countries | ✓ | ✓ |  | ✓ |  |
| Reimbursement for treatment varies across regions, creating challenges in standardizing national treatment practice |  |  |  | ✓ |  |
| Issues of a burden of hospital litigation associated with CDI |  | ✓ |  |  |  |
| Education and awareness raising for patients/care givers |  |  |  |  |  |
| Patient-related issues such as stigma, disgust and embarrassment or low awareness of and understanding of CDI symptoms can be a barrier to timely diagnosis, leading to patients delaying seeking help from a healthcare professional or not providing all the information about their symptoms. | ✓ |  |  | ✓ |  |
| A scarcity of public health campaigns (national and regional) about CDI symptoms and the importance of seeking care can also impact on access to the right care at the right time and place. It can also impact on resorting to treatments for which sufficient evidence may be lacking, such as probiotics. |  |  |  | ✓ |  |
| There is limited evidence on the impact of CDI from the patient or care-giver perspectives, and this is an area that requires further research. |  |  |  | ✓ |  |
| Education and awareness raising for clinicians |  |  |  |  |  |
| Clinician awareness and knowledge of *C. difficile* diagnostic, treatment and referral processes can be relatively low, especially in primary care and amongst some specialist clinicians such as surgeons, partly due to CDI not being in the medical curriculum (or having not been there in the past) and lack of awareness of guideline updates. | ✓ | ✓ |  | ✓ | ✓ |
| There can be risks to clinicians not interpreting test results correctly and treating a patient in cases where *C. difficile* has been detected but is not toxigenic. | ✓ | ✓ | ✓ | ✓ | ✓ |
| Impact of COVID-19 on service delivery |  |  |  |  |  |
| FMT services were stopped at the start of the pandemic, presenting challenges to delaying patient care | ✓ | ✓ | ✓ |  |  |
| Risk of CDI being deprioritized as efforts are focused on dealing with the pandemic | ✓ | ✓ |  |  | ✓ |
| Patients may have avoided seeking healthcare due to fears of contracting COVID-19 |  |  |  |  |  |
